# Complex Crystal Structure Determination of Hsp90^N^-NVP-AUY922 and *In Vitro* Anti-NSCLC Activity of NVP-AUY922

**DOI:** 10.3389/fonc.2022.847556

**Published:** 2022-02-24

**Authors:** Chun-Xia He, You Lv, Meng Guo, Huan Zhou, Wei Qin, Dong Zhao, Hui-Jin Li, Lu Xing, Xin Zhou, Peng-Quan Li, Feng Yu, Jian-Hua He, Hui-Ling Cao

**Affiliations:** ^1^ Xi’an Key Laboratory of Basic and Translation of Cardiovascular Metabolic Disease, Shaanxi Key Laboratory of Ischemic Cardiovascular Disease, Institute of Basic & Translational Medicine, Xi’an Medical University, Xi’an, China; ^2^ College of Bioresources Chemical and Materials Engineering, Shaanxi University of Science & Technology, Xi’an, China; ^3^ College of Pharmacy, Shaanxi University of Chinese Medicine, Xianyang, China; ^4^ Shanghai Synchrotron Radiation Facility, Shanghai Advanced Research Institute, Chinese Academy of Sciences, Shanghai, China; ^5^ Institute for Advanced Studies, Wuhan University, Wuhan, China

**Keywords:** NVP-AUY922, Hsp90^N^, non-small-cell lung cancer (NSCLC), complex crystal structure, molecular interaction, drug design

## Abstract

New targeted chemotherapy agents greatly improved five-year survival in NSCLC patients, but which were susceptible to drug resistance. NVP-AUY922, terminated in phase II clinical trials, exhibited promising anti-NSCLC (non-small-cell lung cancer) activity targeting to Hsp90^N^ (heat shock protein), which demonstrated advantages in overcoming drug resistance as a broad-spectrum anti-cancer target. It was expected to develop novel anti-NSCLC drugs to overcome drug resistance by the structural optimization of NVP-AUY922. However, the absence of high-resolution complex crystal structure of Hsp90^N^-NVP-AUY922 blocked the way. Herein, 1.59 Å-resolution complex crystal structure of Hsp90^N^-NVP-AUY922 (PDB ID 6LTI) was successfully determined by X-ray diffraction. Meanwhile, there was a strong binding capability between NVP-AUY922 and its target Hsp90^N^ verified by TSA (ΔTm, −15.56 ± 1.78°C) and ITC (*K*_d_, 5.10 ± 2.10 nM). Results by the complex crystal structure, TSA and ITC verified that NVP-AUY922 well accommodated in the ATP-binding pocket of Hsp90^N^ to disable the molecular chaperone activity of Hsp90. Therefore, NVP-AUY922 exhibited approving inhibitory activity on NSCLC cell line H1299 (IC_50_, 2.85 ± 0.06 μM) by inhibiting cell proliferation, inducing cell cycle arrest and promoting cell apoptosis. At the basis of the complex crystal structure and molecular interaction analysis, thirty-two new NVP-AUY922 derivatives were further designed, and among which twenty-eight new ones display enhanced binding force with Hsp90^N^ by molecular docking evaluation. The results would promote anti-NSCLC new drug development to overcome drug resistance based on the lead compound NVP-AUY922.

**Graphical Abstract:**
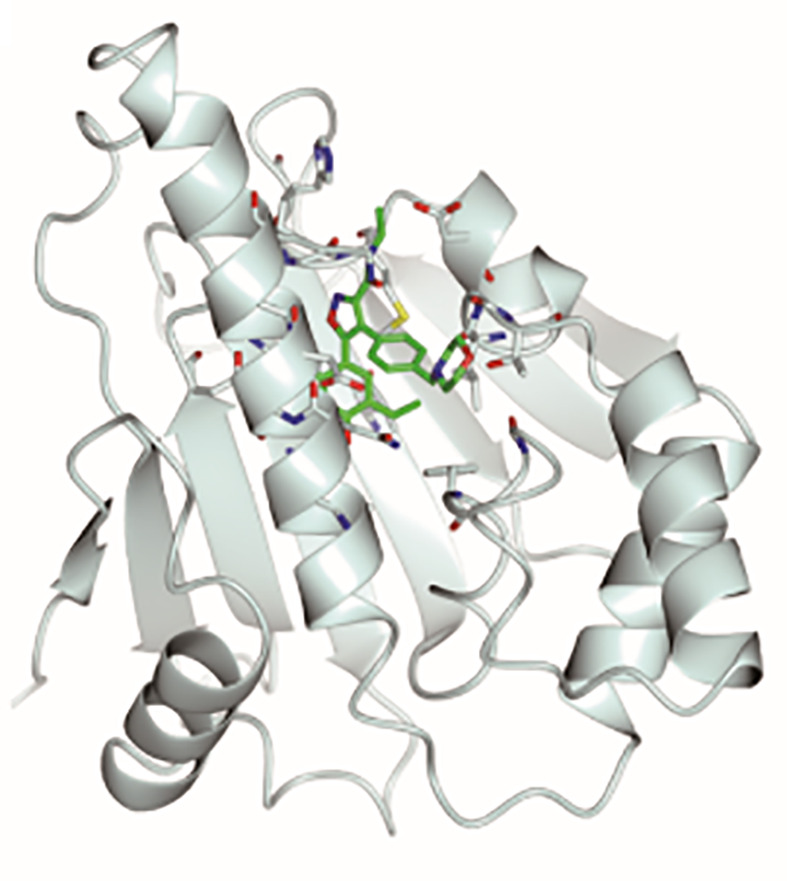
Stereoscopic images of complex crystal structure of Hsp90N-NVP-AUY922. A high-resolution complex crystal structure of Hsp90N-NVP-AUY922 was determined by X-ray diffraction (resolution limit 1.59 Å, PDB ID 6LTI), which suggested that NVP-AUY922 perfectly bound in the N-terminal ATP-binding pocket of Hsp90 to disable its molecular chaperone function, therefore suppressed or killed cancer cells.

## Highlights

1. A high-resolution complex crystal structure of Hsp90N-NVP-AUY922 was successfully determined by X-ray diffraction.
2. NVP-AUY922 well accommodated in the ATP-binding pocket to disable the molecular chaperone function of Hsp90N to inhibit cancer cells verified by the complex crystal structure, thermal shift assay (TSA) and isothermal titration calorimetry (ITC).
3. Thirty-two new NVP-AUY922 derivatives were designed and twenty-eight new ones exhibited increased binding force with the target Hsp90N, which was proven a feasible scheme by molecular docking evaluation.
4. NVP-AUY922 exhibited favourable *in vitro* anti-NSCLC activity.

## Introduction

Lung cancer remained the top mortality cancer causing 1.8 million deaths (18%) among 10.0 million deaths for cancers worldwide in 2020 (1). Lung cancer is therapeutically divided into non-small-cell lung carcinoma (NSCLC) and small cell lung cancer (SCLC), in which NSCLC covers 80% of patients (2). Recently, targeted therapies for lung cancer have been made remarkable progress based on the genomic studies on the subtypes of NSCLC, leading to the improvement of clinical outcomes for NSCLC, but there is still a low 5-year survival rate of less than 20% and susceptible to drug resistance (3). As is well known, cancer cells carry mutant genes and proteins to survive from therapeutic toxins (4). Thus, heat shock protein 90 (Hsp90) inhibitors with non-targeted therapies with broad-spectrum effects demonstrated advantages in overcoming drug resistance, which would be another choice for NSCLC patients with drug resistance.

Hsp90 inhibitors resulted in unfolded and unmatured multiple important client proteins to be captured and degraded by the proteasome by inhibiting molecular chaperone function of Hsp90, therefore suppressed cancer cells (5–7). Hsp90 inhibitors were designed targeting to three domains of Hsp90 monomer that consequently typed as Hsp90^N^ inhibitors, Hsp90^M^ inhibitors, and Hsp90^C^ inhibitors. Hsp90^N^ inhibitors have been a hot spot against tumor cells *in vitro* and *in vivo* (8–12) and many papers and patents focused on Hsp90^N^ inhibitors (13–15). NVP-AUY922, a small molecule inhibitor for Hsp90^N^, is a resorcinol derivative based on the structure of 4,5-diarylisoxazole scaffold, and displayed favorable anti-cancer activity *in vitro* and *in vivo* (16). NVP-AUY922 was evaluated in a phase I clinical trial concerning solid-organ malignancies (primarily breast, ovarian, and colon cancer), and a phase II clinical trial evaluation in NSCLC patients was also carried out by grouping to epidermal growth factor receptor (EGFR) mutations, KRAS proto-oncogene GTPase (KRAS) mutations, anaplastic lymphoma kinase (ALK) rearrangement, and wild-type NSCLC. In phase I clinical trial, major side effects were dose-limiting toxicities, namely, visual symptoms and diarrhea. In phase II clinical trial, NVP-AUY922 exhibited high efficacy in the ALK rearrangement and EGFR mutations. Unfortunately, further development of AUY922 was stopped by Novartis in December of 2014 on account of the failure in meeting its endpoint of complete and partial response of phase II clinical trial in NSCLC patients (7, 17–21).

In brief, NVP-AUY922 was a promising inhibitor for anti-NSCLC new drug development and it was expected to develop novel anti-NSCLC drugs by structural improvement of NVP-AUY922. The absence of high-resolution complex crystal structure of Hsp90^N^-NVP-AUY922 blocked the way. Herein, we successfully determined a high-resolution complex crystal structure (1.59 Å) of Hsp90^N^-NVP-AUY922 by X-ray diffraction (XRD). The molecular interaction mechanism of Hsp90^N^-AUY922 was explored based on the complex crystal structure, thermal shift assay (TSA) and isothermal titration calorimetry (ITC). The findings would promote novel anti-NSCLC drug development to overcome drug resistance based on the lead compound NVP-AUY922.

## Materials and Methods

### Materials and Methods

Human NSCLC cell line H1299 was acquired from the AOLUKEJI (Shanghai, China). The medium RPMI 1640 and fetal bovine serum were purchased from Gibco-BRL (Gaithersburg, MD, USA).

Tris, Tris–HCl, sodium acetate (NaAc), magnesium chloride (MgCl_2_), dimethylsulfoxide (DMSO) and PEG4000 were all from Sigma-Aldrich Corp. (St. Louis, MO, USA). Crystal loop was supplied by Hampton Research Corp. (Aliso Viejo, CA, USA). Sodium chloride (NaCl), glycerol, hydrochloric acid (HCl), sodium hydroxide (NaOH) and ethyl alcohol were obtained from the Xi’an Chemical Reagent Company (Xi’an, China). NVP-AUY922 was from Target Molecule Corp. (Boston, MA, USA). DMSO was used to dissolve NVP-AUY922 and double distilled water (ddH_2_O) was used for other chemicals.

### Cell Viability Assay

First of all, quantitative IC_50_ value was determined. RPMI 1640 with 10% (v/v) fetal bovine serum was used to cultured NSCLC cell line H1299 cells at 37°C in a humidified 5% CO_2_ atmosphere incubator (Thermo Fisher Scientific Inc., Waltham, MA, USA). Briefly, cells were seeded in 96-well plates at 4 × 10^3^ per well in triplicates and were treated with NVP-AUY922 (100, 10, 1, 0.1, 0.01 μM) for 72 h after incubating for 24 h. OD450 was then detected after 2.5 h adding CCK-8 (7Sea, Shanghai, China) by ELISA plate reader (BioTek Instruments, USA).

Cell viability assay was followed and performed. H1299 cells were placed in 96-well plates as described above. Afterwards, cells were treated with 4 × 2.85 μM NVP-AUY922 for 24, 48, and 72 h. After incubating with CCK-8 for 2.5 h, OD_450_ was valued to analyze the cell viability.

### Cell Cycle Analysis

To monitor effects of NVP-AUY922 on cell cycle progression, cells were seeded in 6-well plates at 3 × 10^5^ per well. After treating with 4 × 2.85 μM NVP-AUY922, or the same amount of DMSO for 72 h at 37°C, cells were harvested and resuspended with 3 ml PBS/ethanol mixture (v:v = 1:2) and then preserved at 4°C. The samples were resuspended with 400 μl buffer after 5 min-centrifugation at 1,000*g* and washing in 2 ml PBS once. Lastly, 50 μl propidium iodide (PI) and the same volume of RNase were then added into the cells to be incubated for 30 min in the dark at 37°C (22). Flow cytometry (Becton Dickinson, NJ, USA) was used to analyze the distribution of cell cycle phases.

### Apoptosis Assay

The apoptosis rate was detected by the Annexin V-FITC/PI Apoptosis Detection Kit (556547, Becton Dickinson, USA) according to the manufacturer’s instructions. Briefly, cells were seeded and incubated with NVP-AUY922 as is mentioned in *Cell Cycle Analysis*. Subsequently, cells were resuspended in 100 μl binding buffer after washing with 2 ml PBS. Ultimately, 5 μl Annexin V-FITC and 10 μl PI were added and incubated at 4°C for 30 min in the dark. Also, flow cytometry was adopted for cell apoptosis analysis.

### Protein Purification and Crystallization

The recombination plasmid, pET28a with the synthesized Hsp90^N^ gene (residues 9–236) was transferred into *E. coli* BL21 (DE3) (TIANGEN Biotech Corp., Beijing, China) for the target Hsp90^N^ expression. The protein was purified by Nickel affinity chromatography and molecular sieve chromatography, as previously reported (23).

For crystallization, NVP-AUY922 was mixed with Hsp90^N^ protein (20 mg/ml) with 5:1 molar ratio to incubate for 30 min at 4°C. The mixture was then centrifuged for 10 min at 3,000*g* to collect the supernate. Mixing with the same volume of precipitant (pH 8.5, 0.1 M Tris–HCl, 0.2 M MgCl_2_, 25% PEG4000) (24) was conducted for 3–7 d at 4°C. Hanging drop vapor diffusion method was used. Co-crystallization was accomplished in a bath circulator controlled incubator (PolyScience 9712, PolyScience, USA).

### Data Collection, Structure Determination and Refinement

Hsp90^N^-NVP-AUY922 complex crystal image of was obtained by a stereomicroscope (M165, Leica Microsystems, Germany). Mounted with cryo-loop (Hampton Research Corp., Aliso Viejo, CA, USA), the complex crystals were flash-frozen in liquid nitrogen for XRD after soaking briefly in the cryoprotectant solution (pH8.5, 0.1 M Tris–HCl, 0.2 M MgCl_2_, 25% PEG4000, and 20–25% glycerol). All data sets were acquired at 100 K on Macromolecular Crystallography Beamline17U1 (BL17U1) using an ADSC Quantum 315r CCD detector at Shanghai Synchrotron Radiation Facility (SSRF, Shanghai, China) (25) and the diffraction data were auto-processed by aquarium pipeline (26). The complex crystal structure was analyzed by molecular replacement method using the PHENIX software (27) according to the reported Hsp90^N^-FS23 (PDB ID, 5CF0) (28). The program Coot was used to rebuild the initial model (29). CCP4MG software was used for graphic reconstruction and molecular interaction analysis (30).

### Thermal Shift Assay (TSA)

The ligand NVP-AUY922, dissolved in DMSO, and the target Hsp90^N^ were mixed at a molar ratio of 1:5 in a 20 μl TSA dye reaction buffer with 20 mM Tris–HCl, 150 mM NaCl and 10% glycerol (pH 7.5). The melting-curve (25 to 95°C with a ramp rate of 1°C/min) was determined on ABI 7500 system (7500, ABI Corp., USA). Fluorescence was stimulated by environmentally-sensitive TSA dye binding to the exposed hydrophobic regions during protein unfolding as it is heated. Binding capacity of protein–ligand can be closely evaluated by the melting temperature (Tm) and melting temperature shift (ΔTm) which was calculated based on the melt curves (31).

### Isothermal Titration Calorimetry (ITC)

ITC was performed to further illustrate molecular interaction mechanism of Hsp90^N^-NVP-AUY922 using an ITC-200 calorimeter (Malvern Instrument Ltd., UK). All measurements were carried out in PBS (pH 7.5) at 25°C. Briefly, fresh-purified Hsp90^N^ protein was concentrated to 50 μM. The ligand NVP-AUY922 was prepared in a concentration of 500 μM. Sample was degassed before use. For each titration in the cell, 2 μl aliquot of NVP-AUY922 was added into Hsp90^N^ solution at a 200 s-interval and stirring speed of 750 rpm. The experimental data was fitted to a bimolecular binding model by adjusting three parameters, namely, stoichiometry (n), enthalpy (Δ*H*°) and association constant (*K*a) using Microcal Origin software embedded in the equipment. The thermodynamic parameters Δ*G*° (free energy) and ΔS° (entropy) were obtained according to the formula, −*RT* ln *K*a = Δ*G*° = Δ*H*° − *T*Δ*S*° (32).

### New NVP-AUY922 Derivatives Design and Molecular Docking Evaluation

Based on the analysis of the Hsp90^N^-NVP-AUY922 crystal structure, TSA and ITC, we designed a series of novel NVP-AUY922 derivatives. Evaluation of the derivatives was performed through molecular docking on software SYBYL-X 2.0 (Tripos Associates Inc., St. Louis, MO, USA).

First, a library of novel NVP-AUY922 derivatives was built and a geometric and force field optimization was carried out over 10,000 times. Second, the protein optimization was performed, such as adding hydrogenation and charge. Lastly, the molecular docking evaluation was performed by Surflex-Dock module of SYBYL software. Further, complex 3D structure reconstruction was simulated by CCP4MG software and molecular interaction mechanism was also analyzed (30).

### Statistical Analysis

All data were exhibited as the mean ± standard deviation (SD). The difference significance between two variables was evaluated by Unpaired Student’s t-test, and *P <*0.05 was considered having statistical significance. Data analysis was performed using GraphPad Prism 5.01 (GraphPad Software, San Diego, USA) and SPSS 13.0 (International Business Machines Corporation, USA). Flow cytometry (Becton Dickinson, NJ, USA) was adopted for cell apoptosis analysis.

## Results

### Anti-NSCLC Activity *In Vitro*

AUY-922, as a positive prodrug control, performed favorable anti-NSCLC activity on NSCLC cell lines H460, A549 and H1975 (33). Herein, the effects of NVP-AUY922 on cell viability, cell cycle progression and cell apoptosis of another NSCLC cell line H1299 were analyzed.

**Figure 1:**
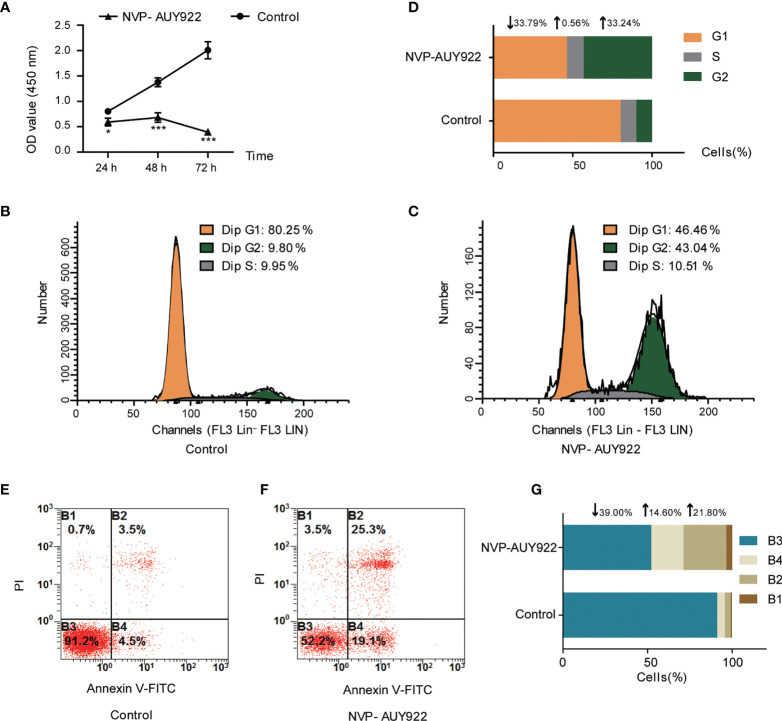
Cell inhibition activity of NVP-AUY922 on NSCLC cell line H1299. **(A)** NVP-AUY922 inhibited H1299 cell viability. NVP-AUY922 significantly suppressed cell viability against H1299 and its inhibition activity increased with elongated treat time. The data was presented as Mean ± SD, *P <0.05, ***P <0.001 (*vs* Control, n = 3) indicated a significant difference versus the control. **(B)** Cell cycle distribution for H1299 treated with DMSO for 72 h. **(C)** Cell cycle distribution for H1299 treated with NVP-AUY922 for 72 h. **(D)** Bar graph for cell percentage in G1, S and G2 phase of H1299 treated with NVP-AUY922 or DMSO for 72 h NVP-AUY922 induced H1299 cell cycle arrest in G2 phase. **(E)** Cell distribution in four quadrants of H1299 treated with DMSO. **(F)** Cell distribution in four quadrants of H1299 treated with NVP-AUY922. **(G)** Bar graph for cell percentage in four quadrants of H1299 treated with NVP-AUY922 or DMSO. Control, H1299 treated with DMSO; NVP-AUY922, H1299 treated with NVP-AUY922. The four quadrants represented as: B1, necrotic cell rate; B2, late apoptotic rate; B3, Normal cell rate; B4, early apoptotic rate. NVP-AUY922 promoted H1299 cell apoptosis. The results indicated that NVP-AUY922 exhibited favorable inhibition activity against H1299 cells by suppressing cell viability, inducing cell cycle arrest and promoting cell apoptosis.

NVP-AUY922 exhibited favorable inhibitory activity against H1299 cells (IC_50_, 2.85 ± 0.063 μM). As shown in **Figure 1A**, NVP-AUY922 significantly inhibited H1299 cell viability at 24 h (*P <*0.01), 48 h (*P <*0.001) and 72 h (*P <*0.0001), and its inhibitory activity was increased with extended treat time.

As can be seen from **Figures 1B**–**D**, after treated with NVP-AUY922 for 72 h, the percentage of H1299 cells in G1, S and G2 phases was obviously changed (NVP-AUY922, G1,↓33.79%; S,↑0.56%; G2,↑33.24% *vs* Control), indicating that when treated with NVP-AUY922, H1299 cells were notably arrested at G2 phase.

Effects of NVP-AUY922 on H1299 cell apoptosis was also analyzed by the annexin V and PI double staining kit by flow cytometry. Briefly, during early apoptosis, Annexin V can bind to phosphatidylserine (PS) that exists on the external leaflet of the plasma membrane. While, early apoptotic cells exclude PI, late apoptotic cells and necrotic cells can be stained positively (34). Cell percentage at different apoptosis stages are shown in **Figures 1E**–**G**. Compared with the control, NVP-AUY922 elevated both the early apoptosis rate and later apoptosis rate of H1299 cells obviously (NVP-AUY922, normal cell rate,↓39.00%; early apoptosis rate,↑14.60%; late apoptosis rate,↑21.80% *vs* Control).

### Molecular Interaction Mechanism Analysis

**Figure 2:**
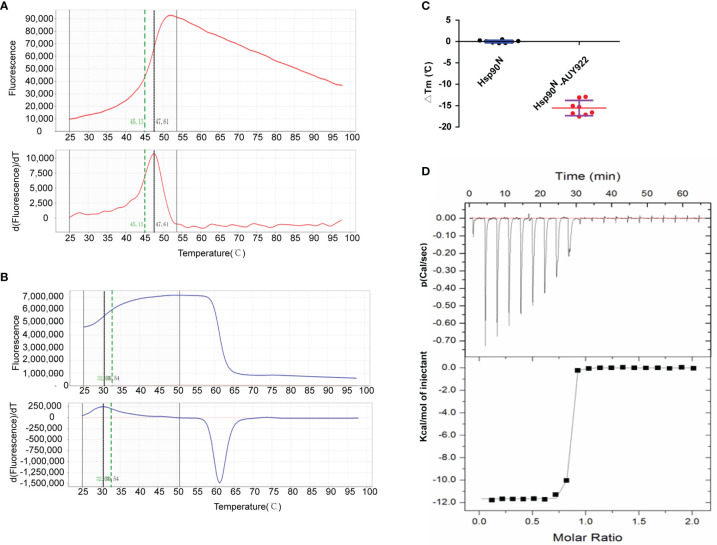
New NVP-AUY922 derivatives design according to complex crystal structure and molecular docking evaluation. **(A)** Design scheme for new NVP-AUY922 derivatives. **(B)** Top ten new derivatives with the highest Total scores evaluated by molecular docking using SYBYL-X 2.0 software. **(C)** Simulated overall 3D structure of Hsp90^N^-A15. Cartoon for Hsp90^N^ and stick for A15. **(D)** Simulated intermolecular force between Hsp90^N^ and A15. For A15, the carbon, nitrogen, oxygen and sulfur atoms of were marked in green, blue, red and yellow, respectively. For Hsp90^N^, the carbon, nitrogen, oxygen and sulfur atoms were marked in cyan, blue, red and yellow, respectively. The red dashed line represents the hydrogen bond. **(E)** Connolly surface of simulated complex 3D structure of Hsp90^N^-A15. **(F)** Molecular structure of A15.

The thermal stability of proteins with or without ligands was detected by TSA according to the Tm and ΔTm calculated by the protein melting curve, which closely reflect the protein–ligand binding force. The affinity between target Hsp90^N^ and ligand can be indicated by the absolute value of ΔTm, while |ΔTm| >3 is usually considered as a favorable ligand. The results showed that after binding with NVP-AUY922, the thermal stability of Hsp90^N^ was shifted by approximately 15.56°C (ΔTm, 15.56 ± 1.78°C), suggesting a strong binding force between the Hsp90^N^ and NVP-AUY922 (**Figures 2A**–**C**).

The dissociation constant of Hsp90^N^-NVP-AUY922 was further determined by ITC at 25°C in PBS. As can be seen in **Figure 2D**, a perfect S-shaped curve was established by nonlinear data fitting. The negative peaks in the raw ITC titration data revealed that the binding process of Hsp90^N^-NVP-AUY922 was exothermic at 25°C. The stoichiometric ratio was 0.78 ± 0.04, suggesting a 1:1 binding mode between Hsp90^N^ and NVP-AUY922. The *K*d values derived here was 5.10 ± 2.10 nM, further indicating a strong binding force of Hsp90^N^-NVP-AUY922, as shown in **Table 1**. The thermodynamic signature was dominated by a large enthalpy change, indicating favorable thermodynamic interactions established in the Hsp90^N^-NVP-AUY922 binding process.

**Table 1:** Dissociation constant and thermodynamic data for the target Hsp90^N^ binding with its ligand NVP-AUY922 at 25°C.

Items	Thermodynamic data (Mean ± SD, n = 4)
N	0.78 ± 0.04
*K*_d_ (nM)	5.10 ± 2.10
Δ*G*_a_ (kcal/mol)	−11.50
Δ*H*_a_ (kcal/mol)	−11.40 ± 0.30
*T*Δ*S*_a_ (kcal/mol)	0.10

**Figure 3:**
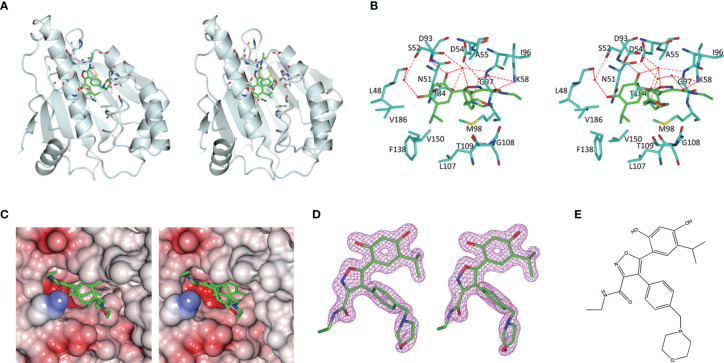
Molecular interaction analysis for NVP-AUY922 and Hsp90^N^ by thermal shift assay (TSA) and isothermal titration calorimetry (ITC). **(A)** Melting curve of protein Hsp90^N^. **(B)** Melting curve of Hsp90^N^ binding with NVP-AUY922. **(C)** ΔTm. (°C) The melting temperature differences of Hsp90^N^ binding with or without NVP-AUY922 (−15.56 ± 1.78°C). Values were presented as Mean ± SD, ***P < 0.001, n = 8. **(D)** ITC plots for Hsp90^N^ binding with its ligand NVP-AUY922. Upper panel: the raw titration data. A constant temperature against time was maintained, interaction heat for injection was given by the area of each peak. Lower panel: the bimolecular data fitting of normalized heats against the molar concentration during Hsp90^N^-NVP-AUY922 binding process. Titrations were performed in PBS (pH 7.5) at 25°C.

As is shown in **Figure 3**, the empty ATP-binding site of Hsp90^N^ showed continuous and strong electron density, which was well matched with NVP-AUY922 molecule structure (**Figures 3A, C, D**). A major part of NVP-AUY922 was buried in the ATP-binding pocket of Hsp90^N^, while the morpholine ring was extending out of the pocket. As shown in **Figure 3B**, the complex hydrogen bond networks were responsible for strong binding force of Hsp90^N^-NVP-AUY922. Three direct hydrogen bonds were formed between NVP-AUY922 and residue D93 (2.7 Å), residue K58 (3.0 Å) and residue G97 (3.0 Å) of Hsp90^N^, respectively. Furthermore, one hydroxy group of the isoxazole ring of NVP-AUY922 formed four water-mediated hydrogen bonds with residues D93, T184, and G97. The other hydroxy group of phenyl ring formed three water-mediated hydrogen bonds with residues L48 and S52. The carbanyl group of carboxamide moiety formed three water-mediated hydrogen bonds with residues K58 and D54. In addition to hydrogen bonds, hydrophobic effects, derived from isopropyl group of the benzene ring in NVP-AUY922 with surrounding residues of Hsp90^N^, also played an important role for their powerful binding force. The results suggested a competitive ATP-binding inhibition of NVP-AUY922 against Hsp90^N^, which inactivated the molecular chaperone function of Hsp90^N^, resulting in inhibiting or killing cancer cells.

### Complex Crystal Structure Determination

Hsp90^N^ protein, expressed in *E. coli* BL21 DE3, was purified by immobilized Ni+ affinity chromatography, and followed by gel filtration chromatography. Hsp90^N^ was co-crystallized with NVP-AUY922 by hanging-drop vapor diffusion method. The complex crystals of Hsp90^N^-NVP-AUY922 were obtained at 4 °C after growing for 3–5 d. Crystals were rhombus, with the average dimension of approximately 120 μm × 70 μm × 50 μm (**Supplementary 1, Figure S1**).

Meanwhile, the complex crystal structure of Hsp90^N^-NVP-AUY922 was determined by molecular replacement method, and Hsp90^N^-FS23 (PDB ID 5CF0) (25) was taken as the analytic model. The coordinates and structure factors have been deposited in PDB (PDB ID 6LTI). Data for collection and refinement statistics are described in **Table 2**. A 1.59 Å resolution limit diffraction data was collected with a final *R_free_* of 0.20 and *R_work_* of 0.18, co-crystal space group of *I* 222, and unit cell parameters of *a* = 66.18 Å, *b* = 89.29 Å, *c* = 99.56 Å; *α* = *β* = *γ* = 90.00°. Residues Glu16–Lys224 were observed in the refined model structure with no electron density for N-terminal residues Asp9–Glu15 and C-terminal residues Glu225–Glu236. The missing residues were conceivable for the N- and C-termini disordered electron density.

**Table 2:** Data processing and refinement for complex crystal of Hsp90^N^-NVP-AUY922.

Diffraction source	BL17U1, SSRF^a^
***Diffraction data***	
Resolution (Å)	50.00–1.59 (1.62–1.59)^a^
Space group	*I* 222
Unit cell parameters	
*a*, *b*, *c* (Å)	66.18, 89.29, 99.56
*α*, *β*, *γ* (°)	90.00, 90.00, 90.00
Wavelength (Å)	0.97890
Total reflections	521,803
Unique reflections	40,044
Redundancy	13.00 (12.10)^a^
Mean I/*σ* (I)	134.80/2.90 (1.90/1.10)
Completeness (%)	99.70 (100.0)^a^
*R_sym_ or R*_merge_ ^a^	0.074 (1.013)^a^
***Refinement data***	
Resolution range (Å)	44.64–1.59
Reflections in working set	38,034
Reflections in test set	2,000
*R*_work_ ^b^/*R*_free_ ^c^ (%)	0.18/0.20
Mean temperature factor (Å^2^)	32.23
Bond lengths (Å)	0.007
Bond angles (°)	0.844

a Data in parentheses are values for the highest-resolution shells.

b R_work_= Σ_hkl_ ||F_obs_ |−|F_calc_||/Σ_hkl_|F_obs_|, F_obs_ are observed structure factors, F_calc_ are calculated structure factors.

c R_free_, calculated similar as R_work_, with 5% of data from a test set excluded from the refinement calculation.

### New NVP-AUY922 Derivatives Design and Molecular Docking Evaluation

**Figure 4:**
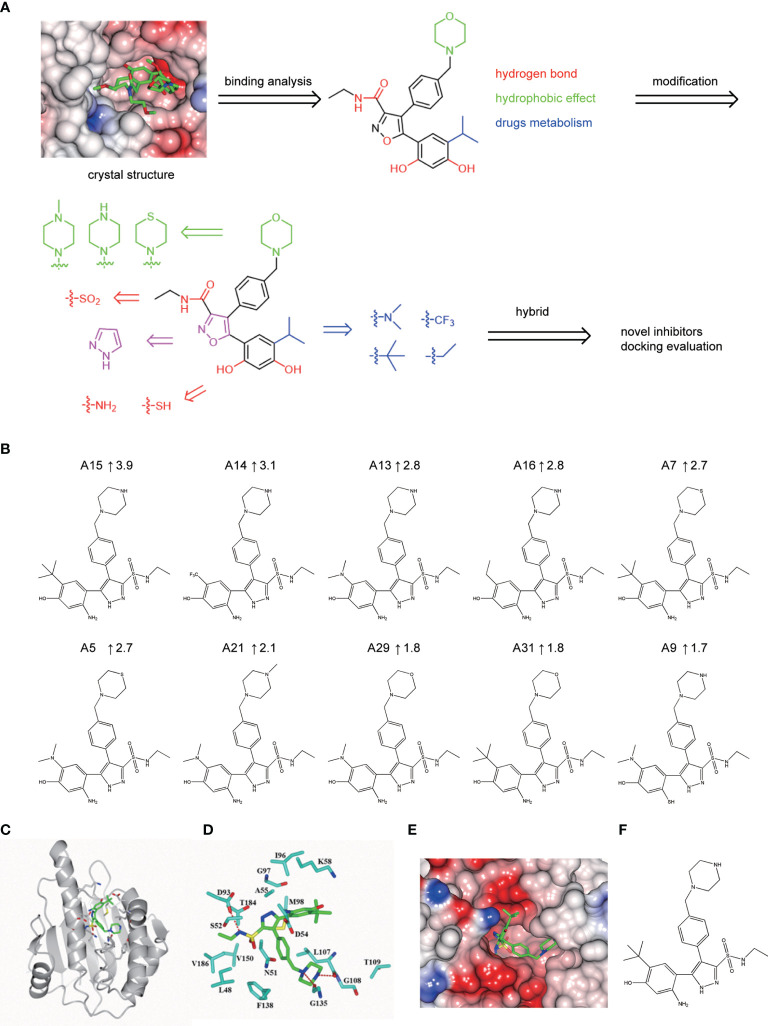
Hsp90^N^-NVP-AUY922 complex crystal structure. **(A)** Overall structural view of Hsp90^N^-NVP-AUY922. Hsp90^N^ and NVP-AUY922 were indicated by cartoon and stick, respectively. **(B)** Intermolecular forces between Hsp90^N^ and NVP-AUY922. For NVP-AUY922, the carbon, nitrogen and oxygen atoms were colored in green, blue and red, respectively. For Hsp90^N^, the carbon, nitrogen, oxygen and sulfur atoms were shown in cyan, blue, red and yellow, respectively. The red dashed line represents the hydrogen bond. The red spheres showed water molecules. **(C)** Distribution of electrostatic potential on Hsp90^N^-NVP-AUY922 crystal structure surface. Red colored surface reflects the negative charge of electrostatic potential, and blue for positive charge. **(D)** Electron density map (2*Fo*-*Fc*) of NVP-AUY922. The map was calculated in the vicinity of NVP-AUY922. **(E)** Molecular structure of NVP-AUY922.

A series of novel NVP-AUY922 derivatives was designed according to the complex crystal structure and molecular interaction analysis, as is shown in **Figure 4A**. For the new derivative design, it was crucial to maintain the special molecular conformation of NVP-AUY922. First, hydrogen bonds played a key role and introduction of hydrophilic groups could be a favorable strategy. For instance, the 2,4-Dihydroxy-5-isopropyl-phenyl ring moiety was varied to an amino group or sulfydryl group, the isoxazole ring was modified to a 1*H*-pyrazole ring and the carbanyl group of formamide group was changed by a sulfonyl group. Second, hydrophobic effects were another specific factor contributed to their affinity. Therefore, transformation of hydrophobic functional groups was considered as appropriate structural modification scheme, followed by isopropyl group on the 2,4-Dihydroxy-5-isopropyl-phenyl ring moiety was replaced by a dimethylamino, trifluoromethyl, ethane or tert-butyl group, respectively. Last, considering the further drug metabolism, a thiomorpholine, piperazine or 1-methyl-piperazine was used to substitute the morpholine ring.

Based on the scheme, thirty-two novel NVP-AUY922 derivatives were successfully designed and evaluated by molecular docking with Total score and Cscore. The molecular structures, Total scores and Total score increments compared with NVP-AUY922 for new derivatives are presented in **Supplementary 2 Table S1**. Total score, evaluates the binding force between target and ligand, is the negative logarithmic value (−log) of the dissociation constant (*K*_d_) of target-ligand, with effects of hydrophobic interaction, polarity, repulsion, entropy change, hydrogen bonds, collision, and so on, to be considered. Higher values indicate stronger binding force of target-ligand. Cscore is a comprehensive score of D-score, PMF-score, G-score, Chem-score, and Total score. The max score is five points. Usually, derivatives with high scores (Total score ≥6, Csore ≥4) were recognized as favorable ligands. Twenty-eight novel NVP-AUY922 derivatives showed increased binding force with the target Hsp90^N^ shown as high Total scores, which manifested a feasible design scheme by molecular docking evaluation. The molecular structures and the Total score increment of top ten new derivatives are shown in **Figure 4B**.

A15 perfomed the top optimal representative (Total score, 11.9). Thereby, simulated complex 3D structure of Hsp90^N^-A15 was built, and analyzed their molecular interaction mechanism in detail. A15 was accommodated appropriately in the ATP-binding pocket of Hsp90^N^ (**Figure 4C**). Compared with NVP-AUY922, the 5-amino-2-tert-butyl-phenol group of A15 was obviously shifted from the interior of cavity to the exterior. Meanwhile, the branched chain of pyrazole ring was moved oppositely from the external space into the internal pocket. Besides, the piperazine ring of A15 was shrunk gently into the pocket (**Figures 4C**–**E**), while the morpholine ring of NVP-AUY922 was extended out of the cavity (**Figure 4A**).

Three hydrogen bonds between A15 and residues D93 (2.9 Å), L107 (2.7 Å) and G135 (3.0 Å) of Hsp90^N^ were formed, respectively. Meanwhile, the piperazine ring together with adjacent aromatic ring formed hydrophobic interactions with residue G135, the pyrazole ring with branched chain arranged hydrophobic contacts with residues S52, M98, and V186, the 5-amino-2-tert-butyl-phenol group formed hydrophobic contacts with residues D54 and A55 (**Figure 4D**). Therefore, the hydrogen bonds and hydrophobic interactions played an important role for the intense binding of Hsp90^N^-A15. The results would provide promising lead compounds for anti-NSCLC new drug development based on NVP-AUY922.

## Discussion

Lung cancer is the top one mortality cancer and NSCLC covers 80% of patients (2). Recently, targeted therapies for lung cancer have been made remarkable progress based on the genomic studies on the subtypes of NSCLC, leading to the improvement of clinical outcomes for NSCLC, but there is still a low 5-year survival rate of less than 20% and susceptible to drug resistance (3). As is well known, cancer cells carry mutant genes and proteins to survive from therapeutic toxins (4). Hsp90, a well-known molecular chaperone, played a crucial role in tumorigenesis and progression. Hsp90 can assist over 280 client proteins in correct folding and conformational maturation, which are widely involved in signal transduction, cell cycle and apoptosis regulation in cancer cells. Therefore, Hsp90 demonstrated advantages in overcoming drug resistance as a broad-spectrum anti-cancer target, which would be another choice for NSCLC patients with drug resistance. Because the activity of chaperone molecule must obtain energy from ATP hydrolysis, Hsp90^N^ inhibitors disabled molecular chaperone function of Hsp90 by competitively suppressing ATP-binding to result in multiple misfolded or immature client proteins that were captured and degraded by the proteasome, cancer cells were thereby suppressed or killed. Therefore, the development of Hsp90 inhibitors has always been a significant tactic for anti-NSCLC drug development (5–9).

NVP-AUY922, a promising second generation Hsp90^N^ inhibitor, exhibited high objective response rate in NSCLC patients with the ALK rearrangement and EGFR mutations. Though NVP-AUY922 was terminated in a phase II clinical trial by Novartis for unsatisfied endpoint of complete and partial response (7, 17–20), NVP-AUY922 was still a promising lead compound for anti-NSCLC new drug development by the structural optimization of NVP-AUY922.

Herein, a 1.59 Å-high-resolution complex crystal structure of Hsp90^N^-NVP-AUY922 was successfully determined by XRD, which made it possible to clearly exhibit the binding mode and molecular interaction in detail between NVP-AUY922 and its target Hsp90^N^. The continuous and clear electron density was presented in the empty ATP-binding pocket of Hsp90^N^ and well matched with NVP-AUY922 molecule structure. Except for hydrophobic interactions, NVP-AUY922 exhibited strong affinity to Hsp90^N^ for complex hydrogen bond networks, namely, direct hydrogen bonds and water-mediated hydrogen bonds. There was still a vast room around NVP-AUY922 in the ATP-binding pocket for further structural optimization.

Crystal structure analysis, TSA and ITC were comprehensively assessed to study the molecular interaction mechanism of Hsp90^N^-NVP-AUY922. Melting temperature (Tm) is a definite parameter for protein under certain conditions which is changed while binding ligands (ΔTm). TSA was applied to detect the protein thermal stability with or without ligands according to the Tm and ΔTm calculated by protein melting curve. Greater affinity was indicated by Greater ΔTm and |ΔTm| >3 is usually deemed as a favorable ligand. There was a strong affinity between the Hsp90^N^ and its ligand NVP-AUY922 (ΔTm, −15.56 ± 1.78°C), suggesting that NVP-AUY922 binding to Hsp90^N^ enhanced the protein thermostability. This was further verified by ITC (*K*_d_, 5.10 ± 2.10 nM). ITC evaluated target-ligand binding force by examining the endothermic and exothermic amount when ligand binding to its target. Smaller the *K*_d_ is, stronger the binding force is. The large enthalpy change indicated favorable thermodynamic interactions established in the Hsp90^N^-NVP-AUY922 binding process. The molecular interaction analysis suggested that NVP-AUY922 can inactivate the molecular chaperone function of Hsp90 by accommodating in the ATP-binding pocket, which took the responsibility for the favorable anti-NSCLC activity of NVP-AUY922. In practice, NVP-AUY922 suppressed cell proliferation (IC_50_, 2.85 ± 0.06 μM), induced cell cycle arresting at G2 phase and accelerated cell apoptosis of the NSCLC cell line H1299.

For further structural optimization, it was crucial to keep the special molecular conformation of NVP-AUY922, which was kept by hydrogen bonds and hydrophobic interactions. The design scheme devoted to promote these beneficial interactions by functional group replacements. Under the precise reference of molecular interaction analysis by complex crystal structure, TSA and ITC of Hsp90^N^-NVP-AUY922, thirty-two new NVP-AUY922 derivatives were designed and molecular docking evaluation showed twenty-eight of them displayed enhanced binding force to the target Hsp90^N^. This also verified a practicable design scheme. A15 as the optimal representative, simulated Hsp90^N^-A15 3D structure was built and detailed molecular interaction mechanism was analyzed. Compared with NVP-AUY922, A15 exhibited increased binding force with Hsp90^N^ and hydrophobic interactions contributed more than hydrogen bonds for Hsp90^N^-A15 binding. The results would promote novel anti-NSCLC drug development to overcome drug resistance based on the lead compound NVP-AUY922.

## Data Availability Statement

The original contributions presented in the study are included in the article/**Supplementary Material**. Further inquiries can be directed to the corresponding authors.

## Author Contributions

H-LC designed the research study. C-XH, YL, MG, H-JL, LX, XZ, and P-QL performed the research. FY, HZ and J-HH provided help and advice on study design. C-XH, WQ, DZ, and P-QL analyzed the data. C-XH, YL, and MG wrote the manuscript. H-LC, FY, and J-HH revised the paper. All authors listed have made a substantial, direct, and intellectual contribution to the work and approved it for publication.

## Funding

This work was funded by the National Natural Science Foundation of China (U1932130), the Program of Shaanxi Provincial Science and Technology Department (2021JQ-786), the Key Program of Shaanxi Provincial Education Department (17JS117), the Program of Shaanxi Administration of Traditional Chinese Medicine (2019-ZZ-ZY009), the Key Program of Weiyang District Bureau of Science, Technology and Industry Information Technology (201928), the Program of Shaanxi Provincial Research Center for the Project of Prevention and Treatment of Respiratory Diseases (2016HXKF05), and the Talent Program of Xi’an Medical University (2016DOC13, 2017GJFY03).

## Conflict of Interest

The authors declare that the research was conducted in the absence of any commercial or financial relationships that could be construed as a potential conflict of interest.

## Publisher’s Note

All claims expressed in this article are solely those of the authors and do not necessarily represent those of their affiliated organizations, or those of the publisher, the editors and the reviewers. Any product that may be evaluated in this article, or claim that may be made by its manufacturer, is not guaranteed or endorsed by the publisher.
