# Supplementary material for: Complex Crystal Structure Determination of Hsp90N-NVP-AUY922 and In Vitro Anti-NSCLC Activity of NVP-AUY922
Source: Front Oncol. 2022 Feb 24;12:847556. doi: 10.3389/fonc.2022.847556 (PMC8907572; doi:10.3389/fonc.2022.847556)
Supplement: Supplementary file 1 [file DataSheet_1.doc]

| 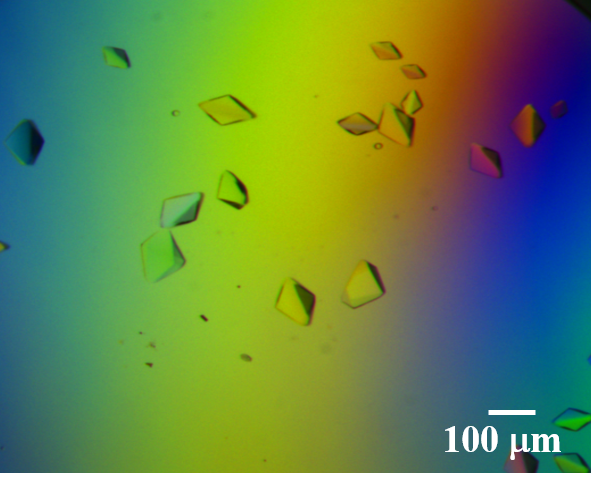 |
| --- |

**Figure A1**. **The complex crystals of Hsp90^N^-NVP-AUY922**. The complex crystals were obtained by the hanging-drop vapor diffusion method at 4℃ for 3～5 d. The average dimension of rhombus crystals was approximately 120 μm×70 μm×50 μm.
