# Supplementary material for: Complex Crystal Structure Determination of Hsp90N-NVP-AUY922 and In Vitro Anti-NSCLC Activity of NVP-AUY922
Source: Front Oncol. 2022 Feb 24;12:847556. doi: 10.3389/fonc.2022.847556 (PMC8907572; doi:10.3389/fonc.2022.847556)
Supplement: Supplementary file 2 [file Table_1.doc]

**Table A1 Molecular docking data of NVP-AUY922 and its new derivatives.**

| **Name** | **Molecular structure** | **Total Score** | **Total Score increment** |
| --- | --- | --- | --- |
| AUY922 | 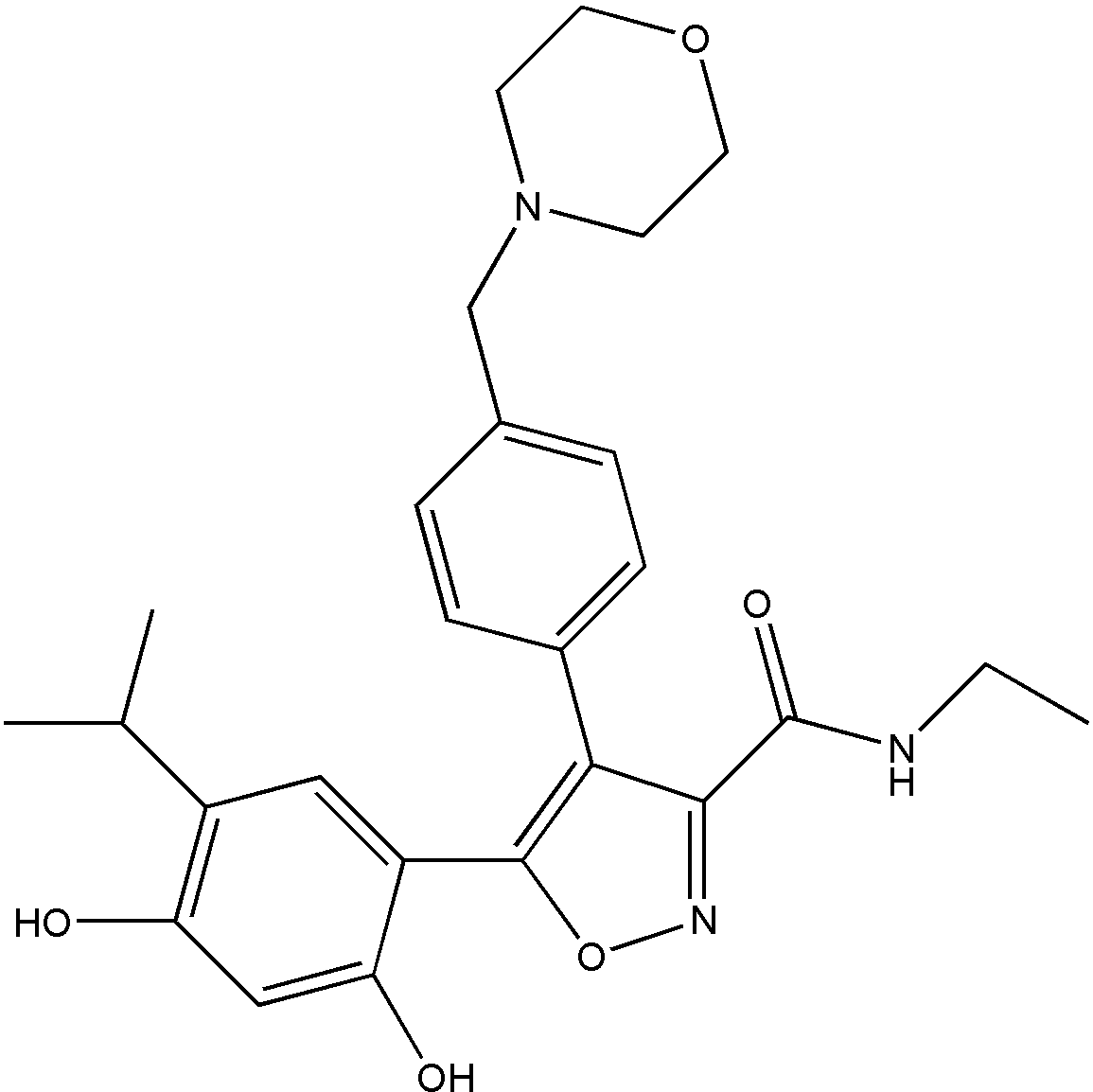 | 8.0 |  |
| A15 | 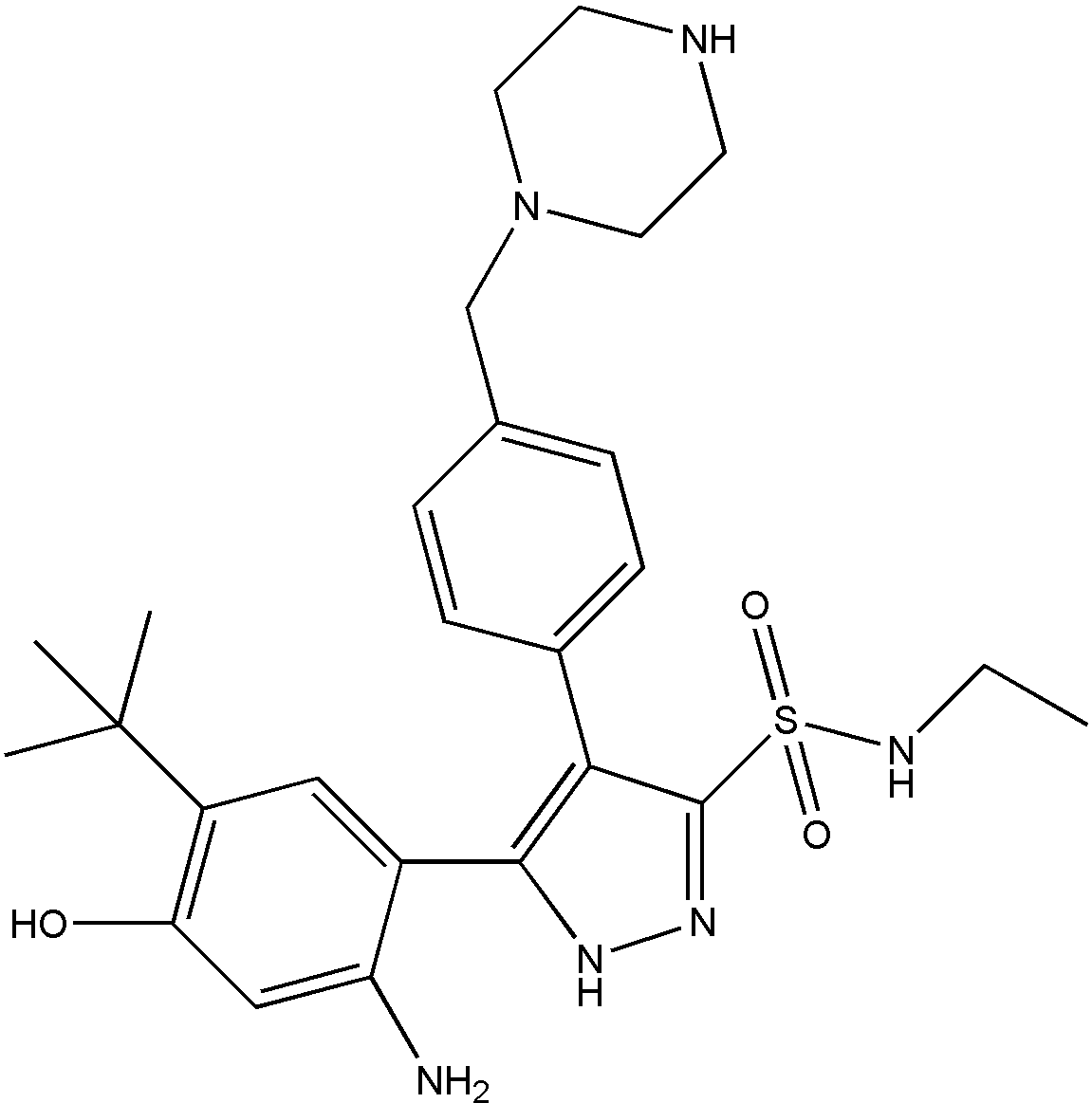 | 11.9 | ↑3.9 |
| A14 | 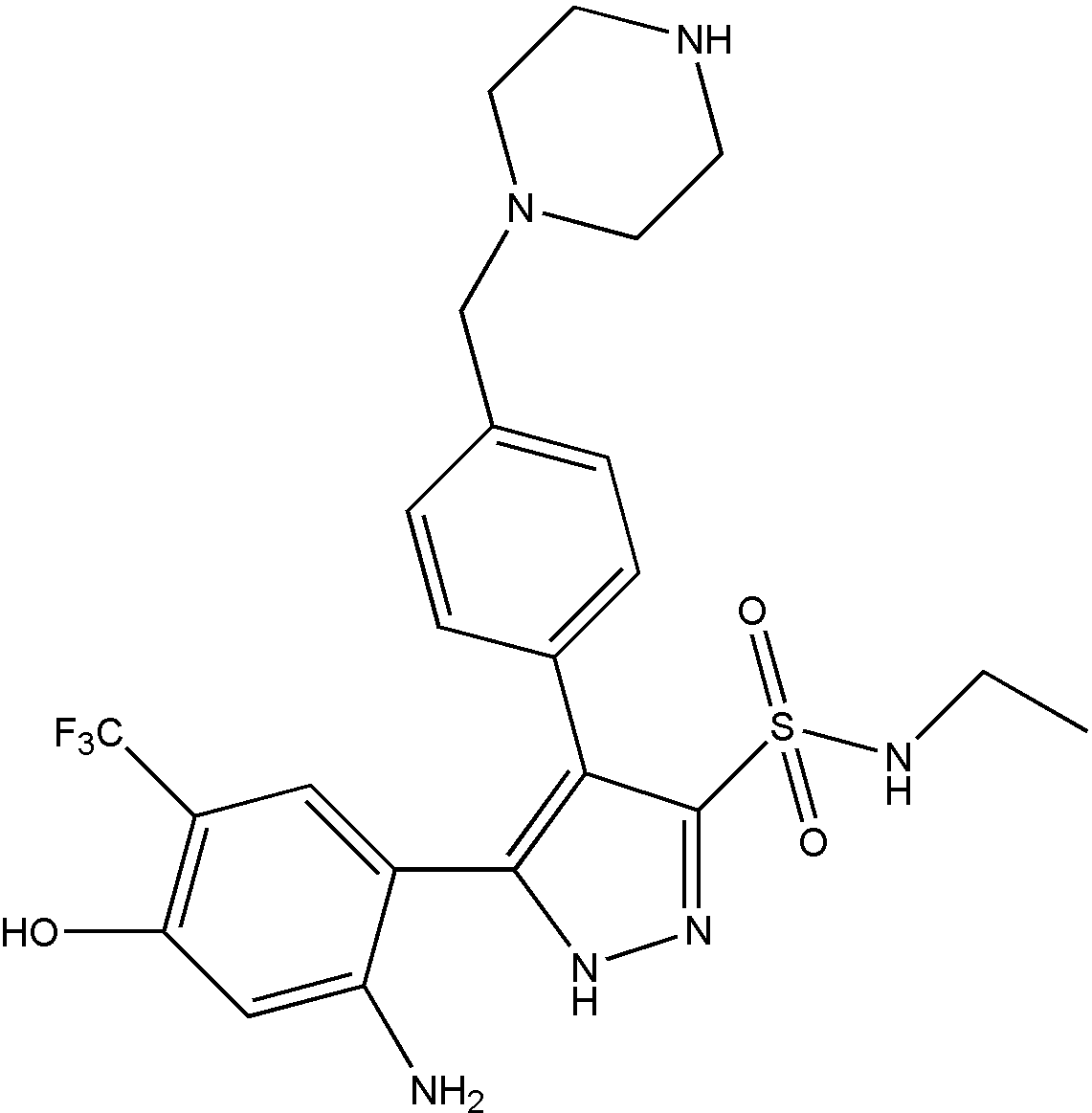 | 11.1 | ↑3.1 |
| A13 | 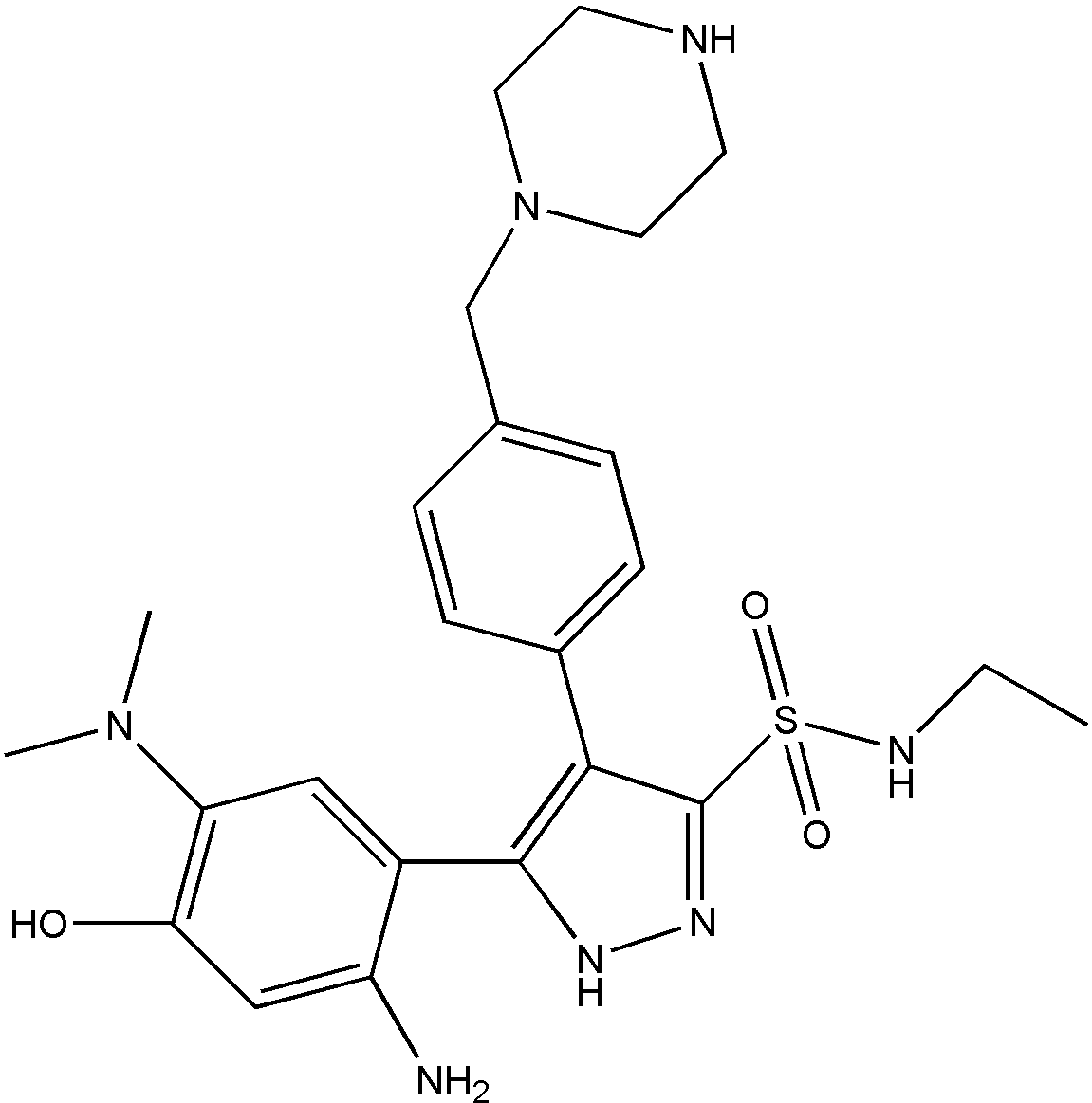 | 10.8 | ↑2.8 |
| A16 | 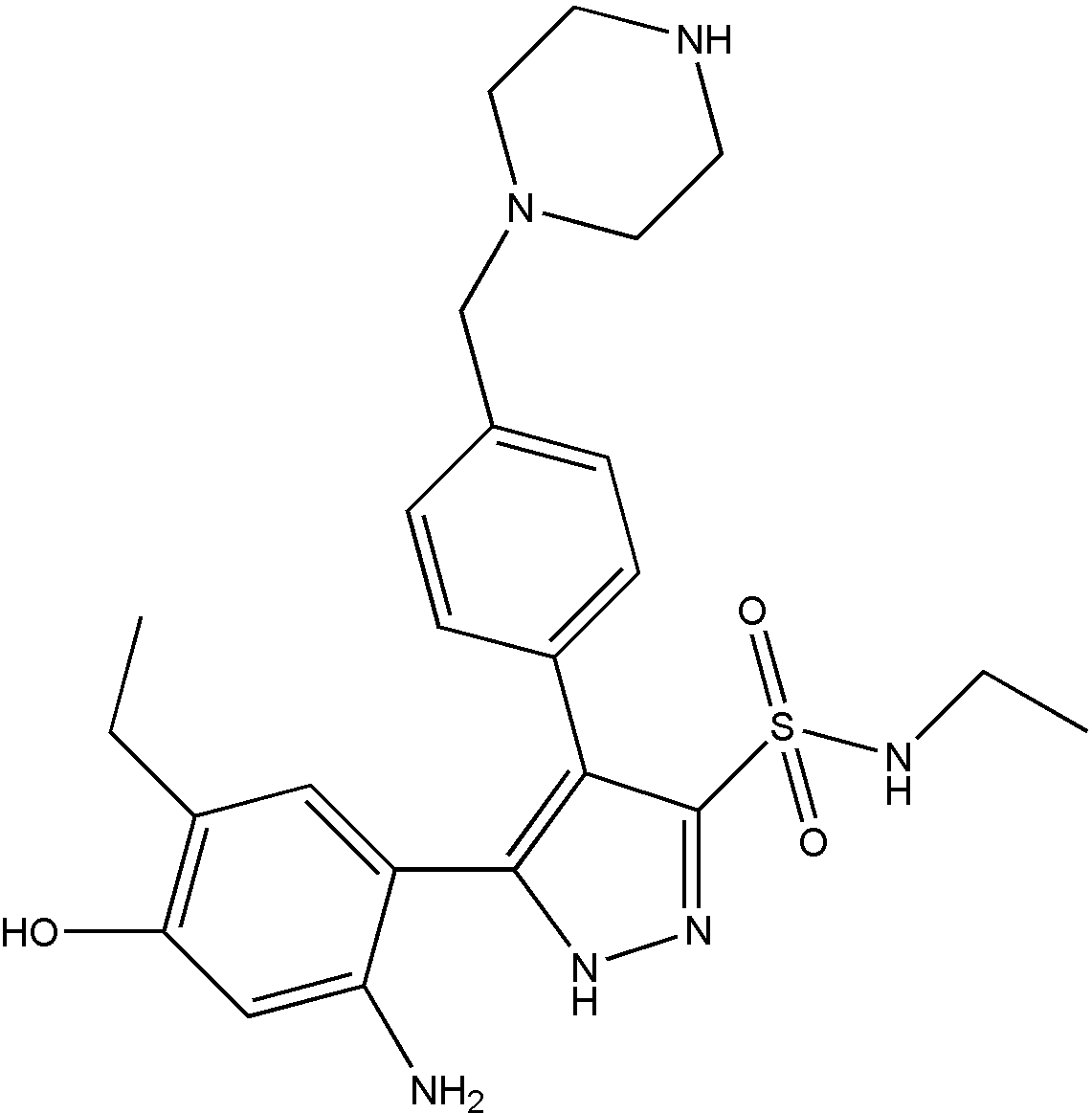 | 10.8 | ↑2.8 |
| A7 | 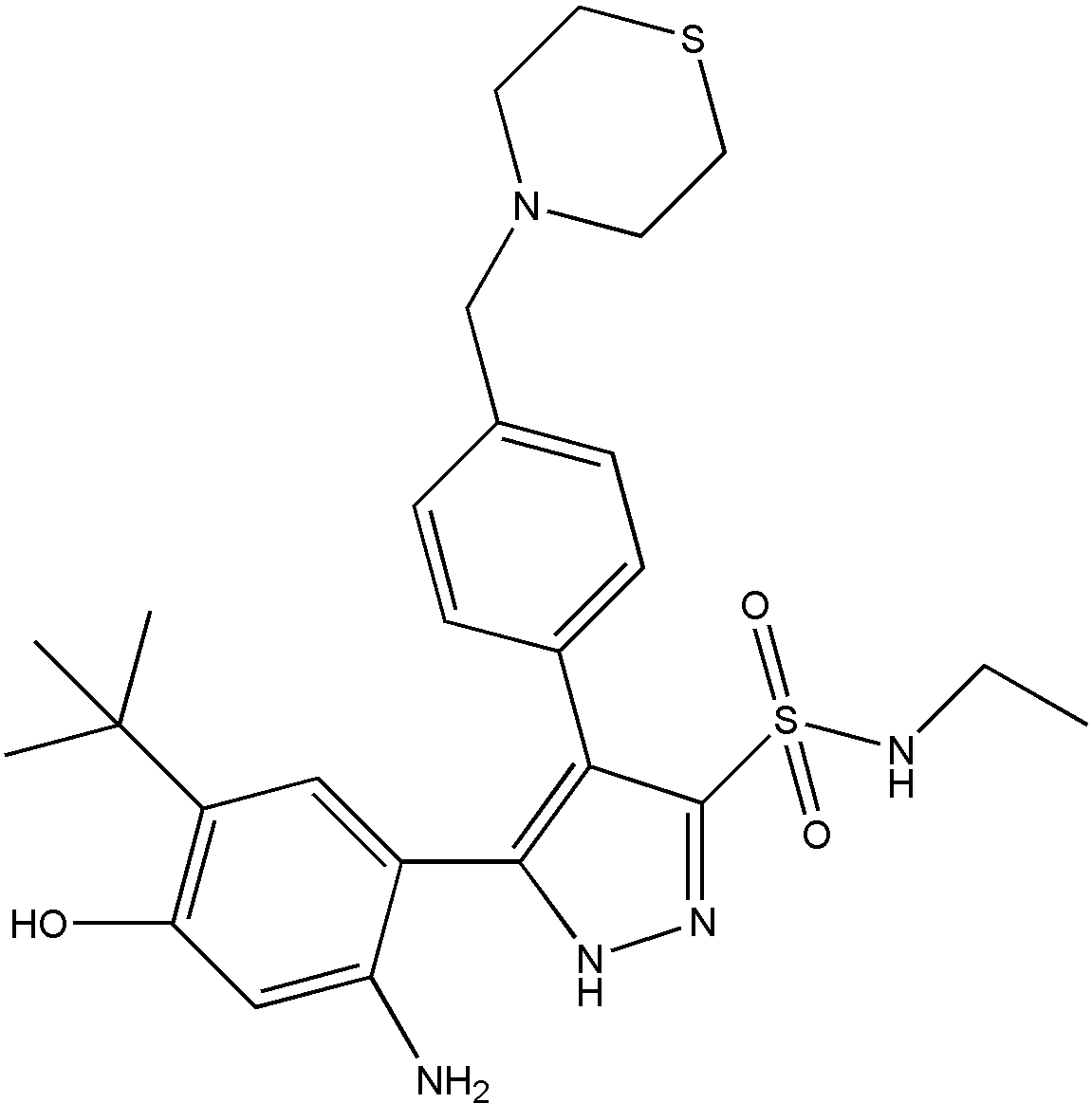 | 10.7 | ↑2.7 |
| A5 | 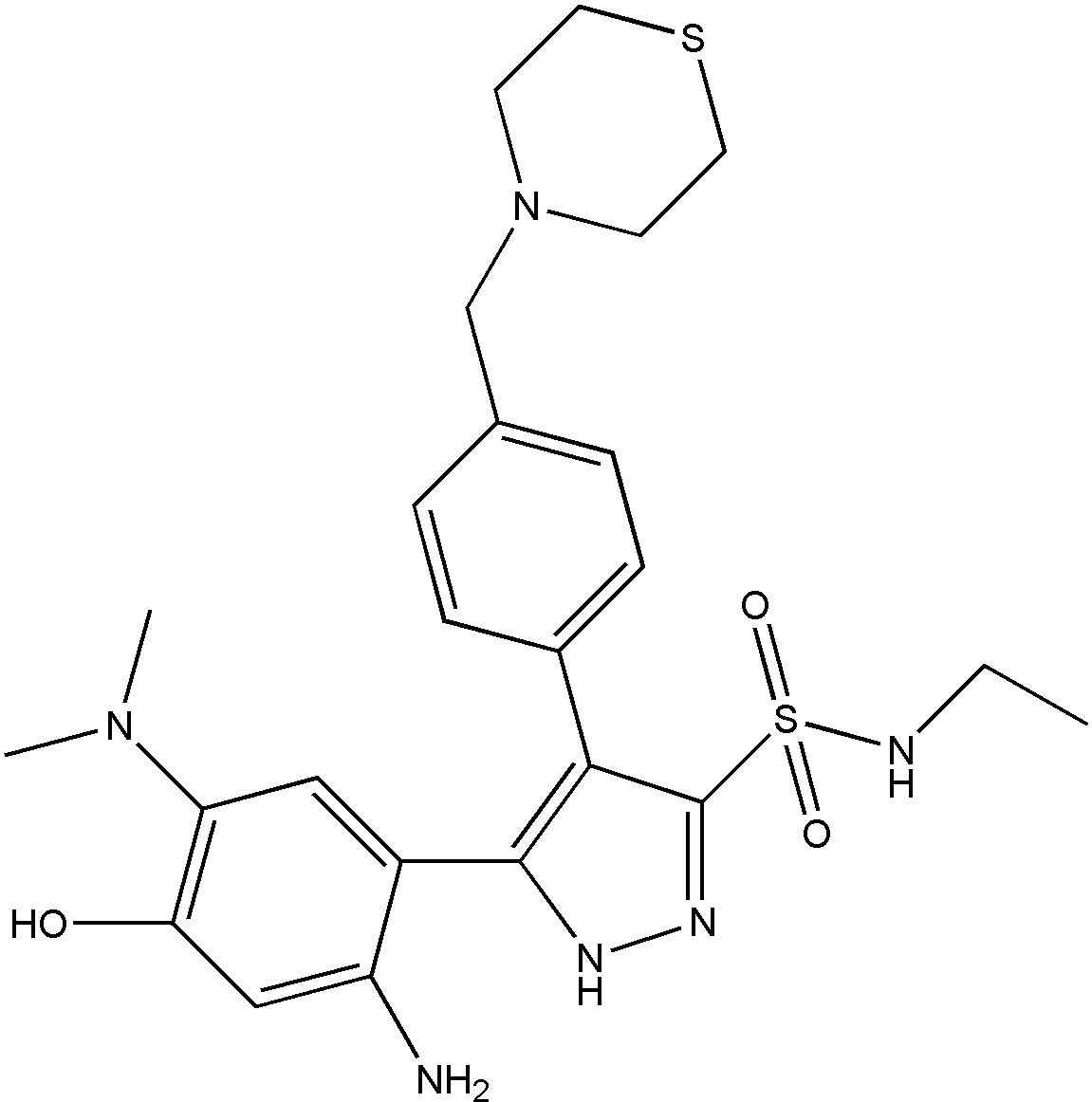 | 10.7 | ↑2.7 |
| A21 | 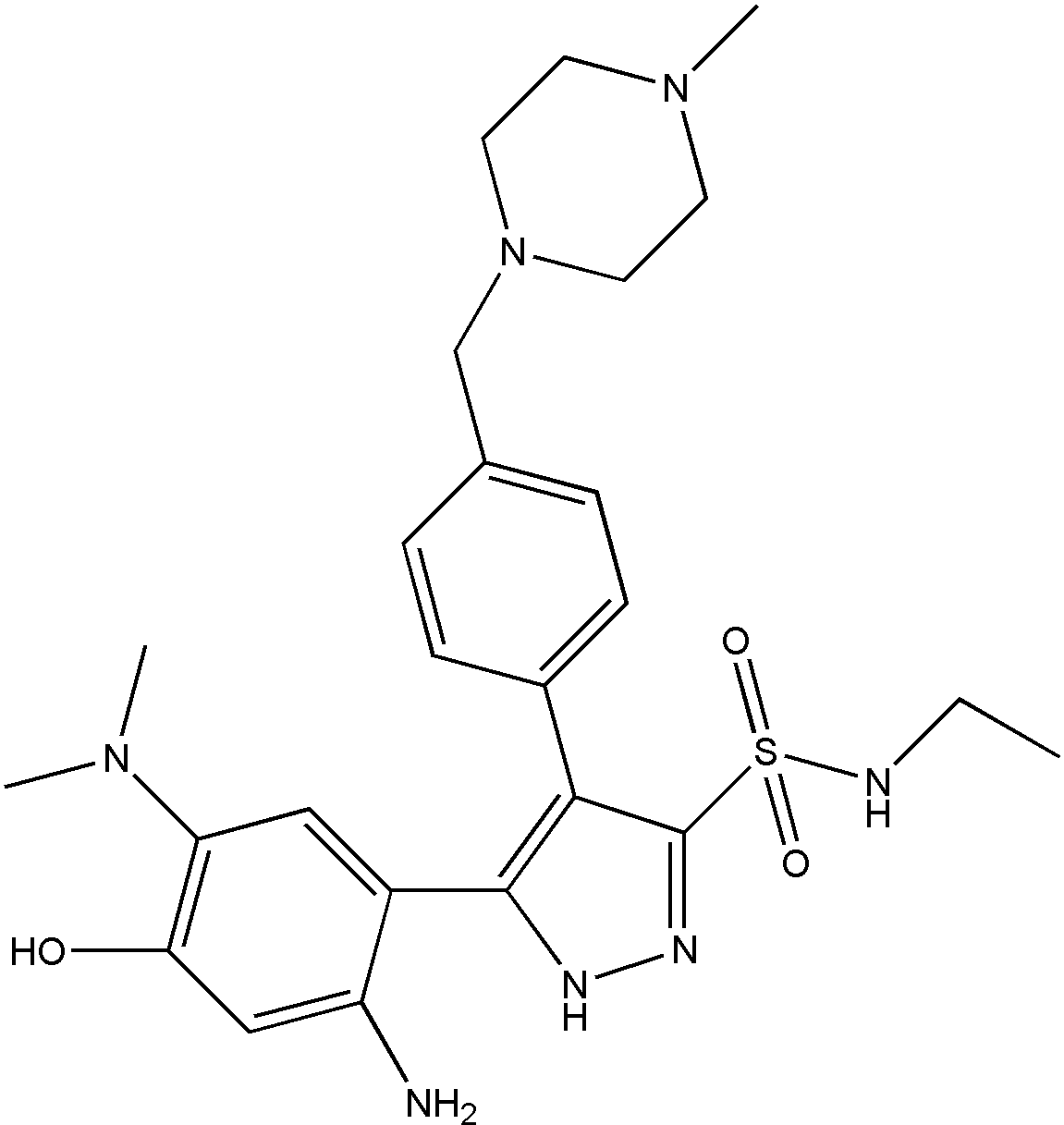 | 10.1 | ↑2.1 |
| A29 | 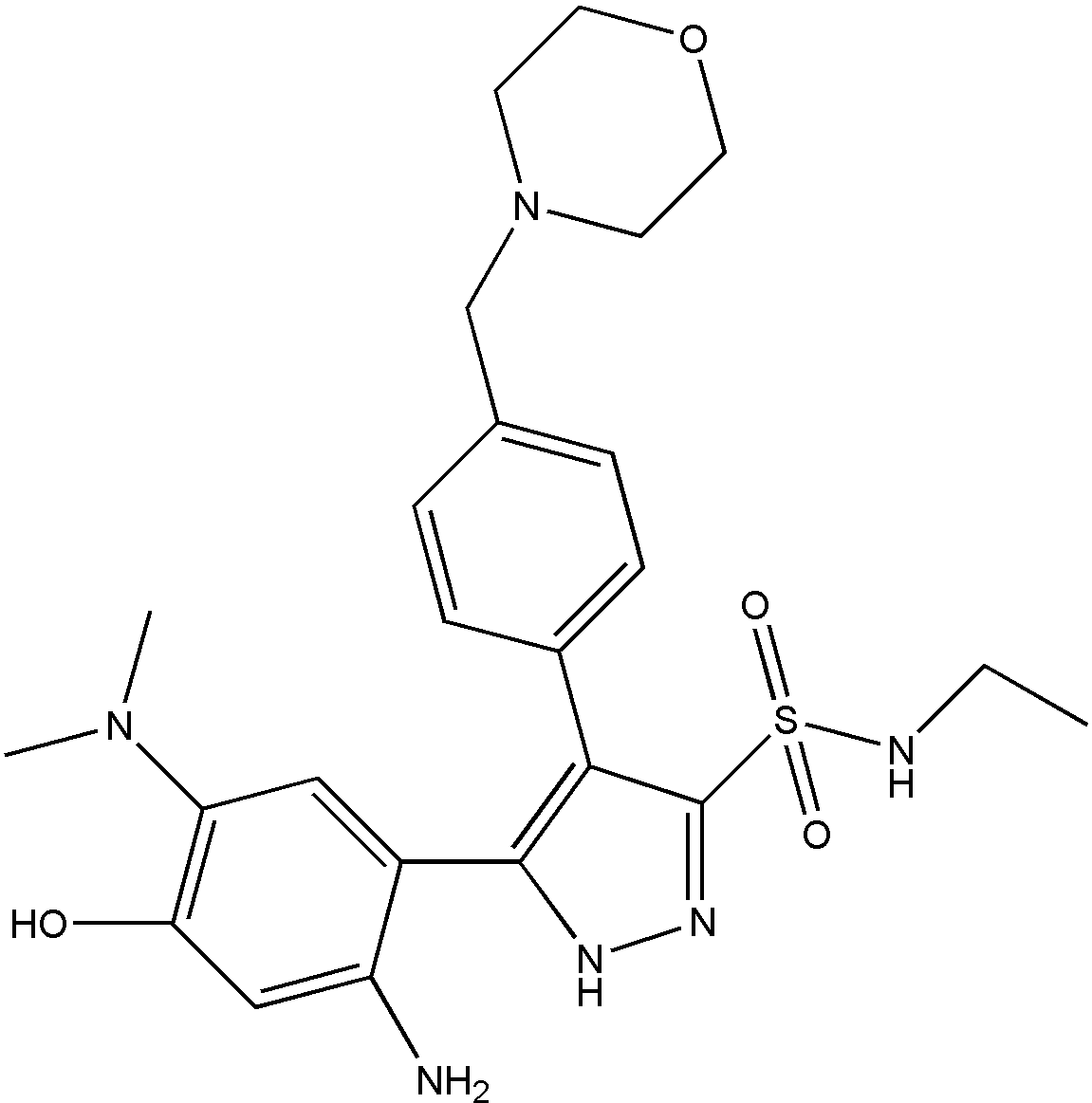 | 9.8 | ↑1.8 |
| A31 | 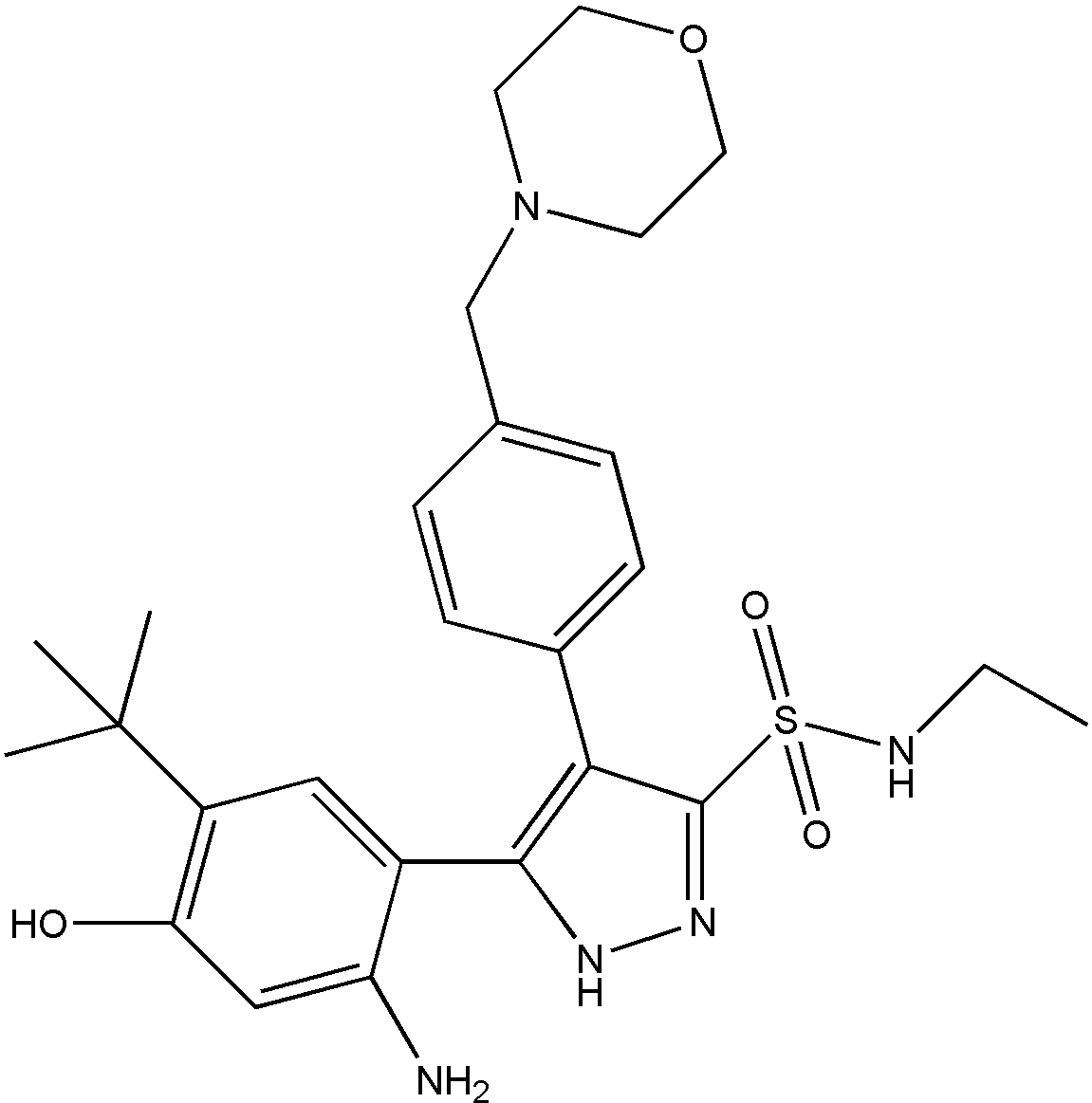 | 9.8 | ↑1.8 |
| A9 | 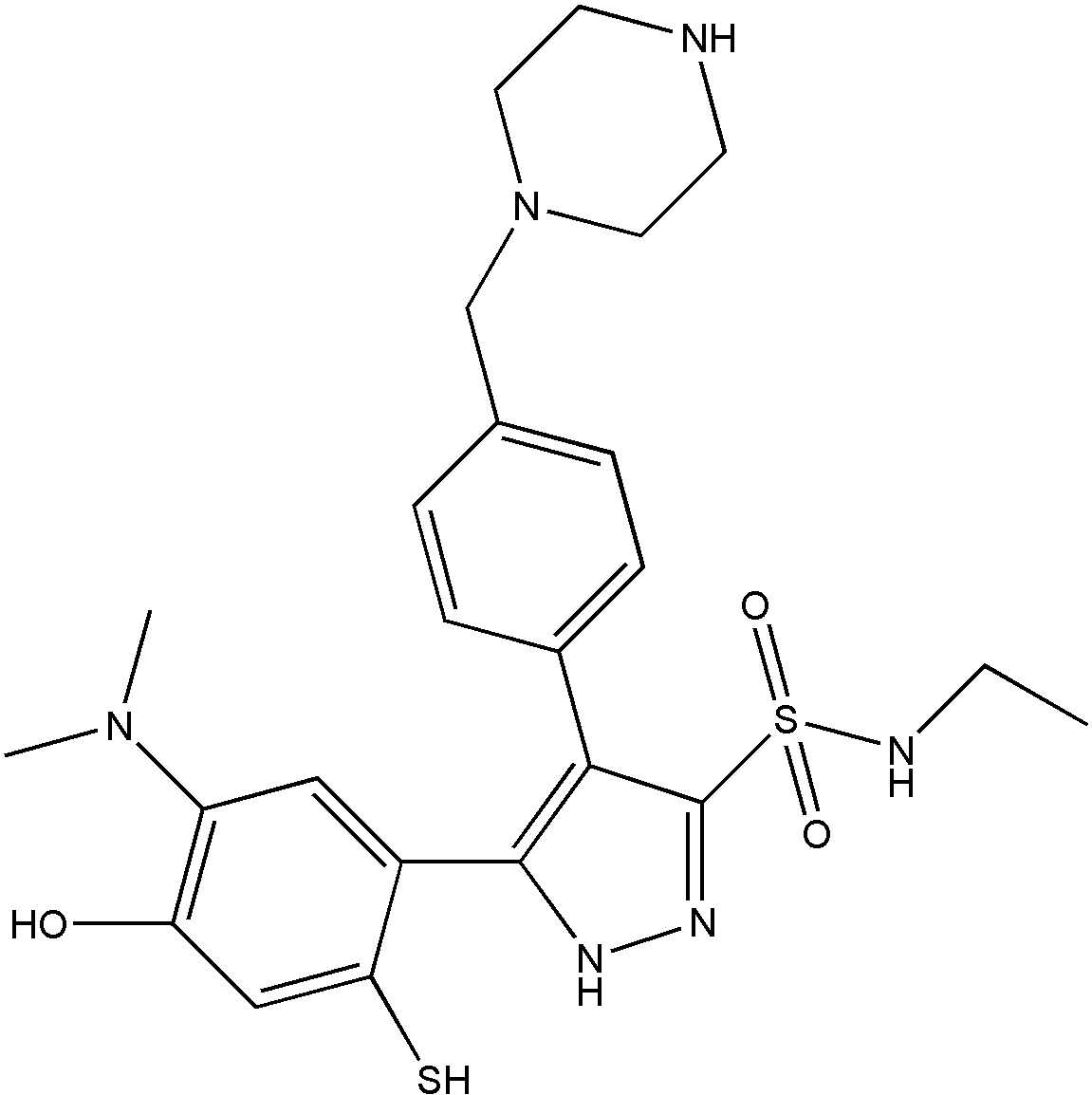 | 9.7 | ↑1.7 |
| A24 | 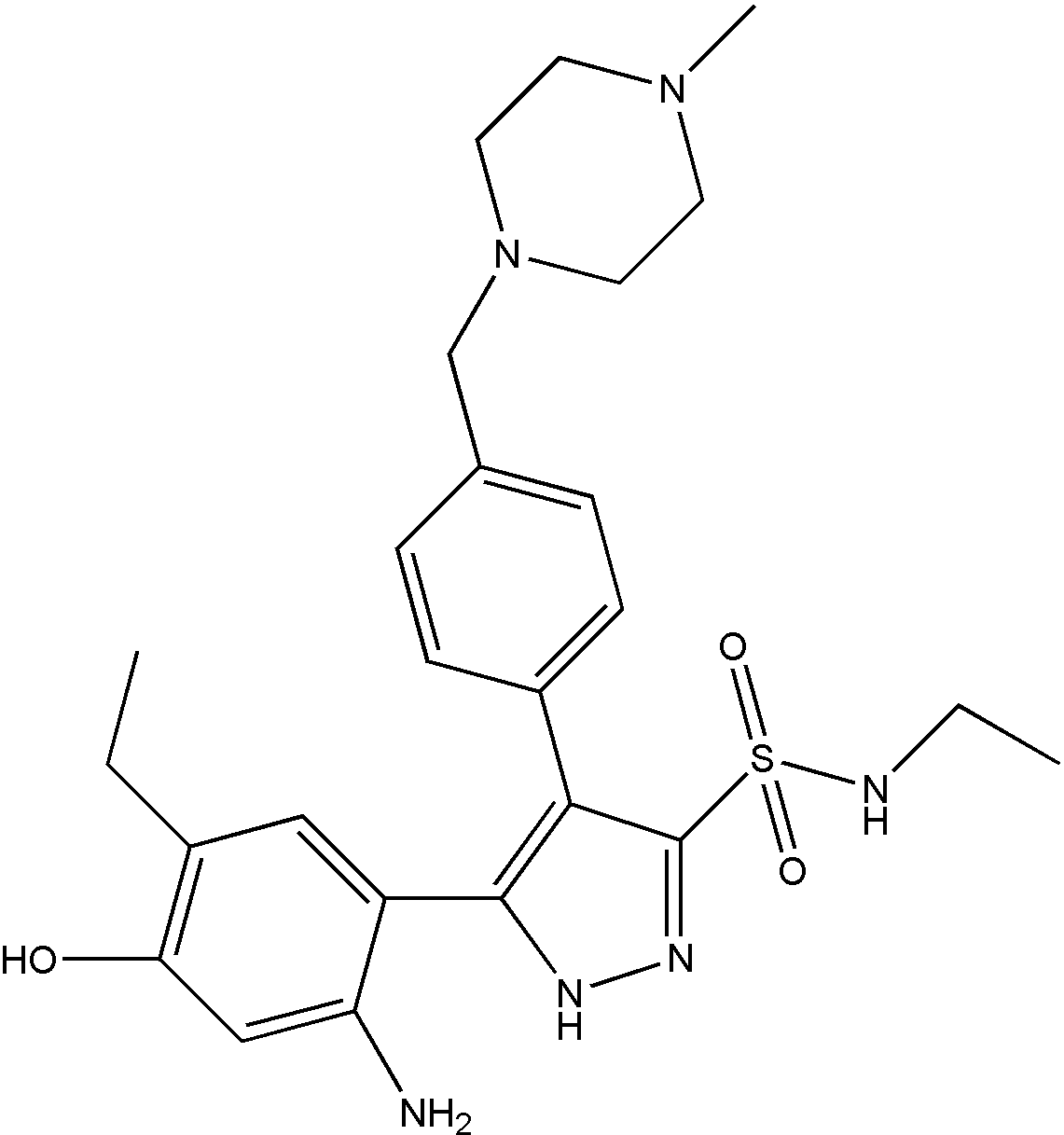 | 9.4 | ↑1.4 |
| A8 | 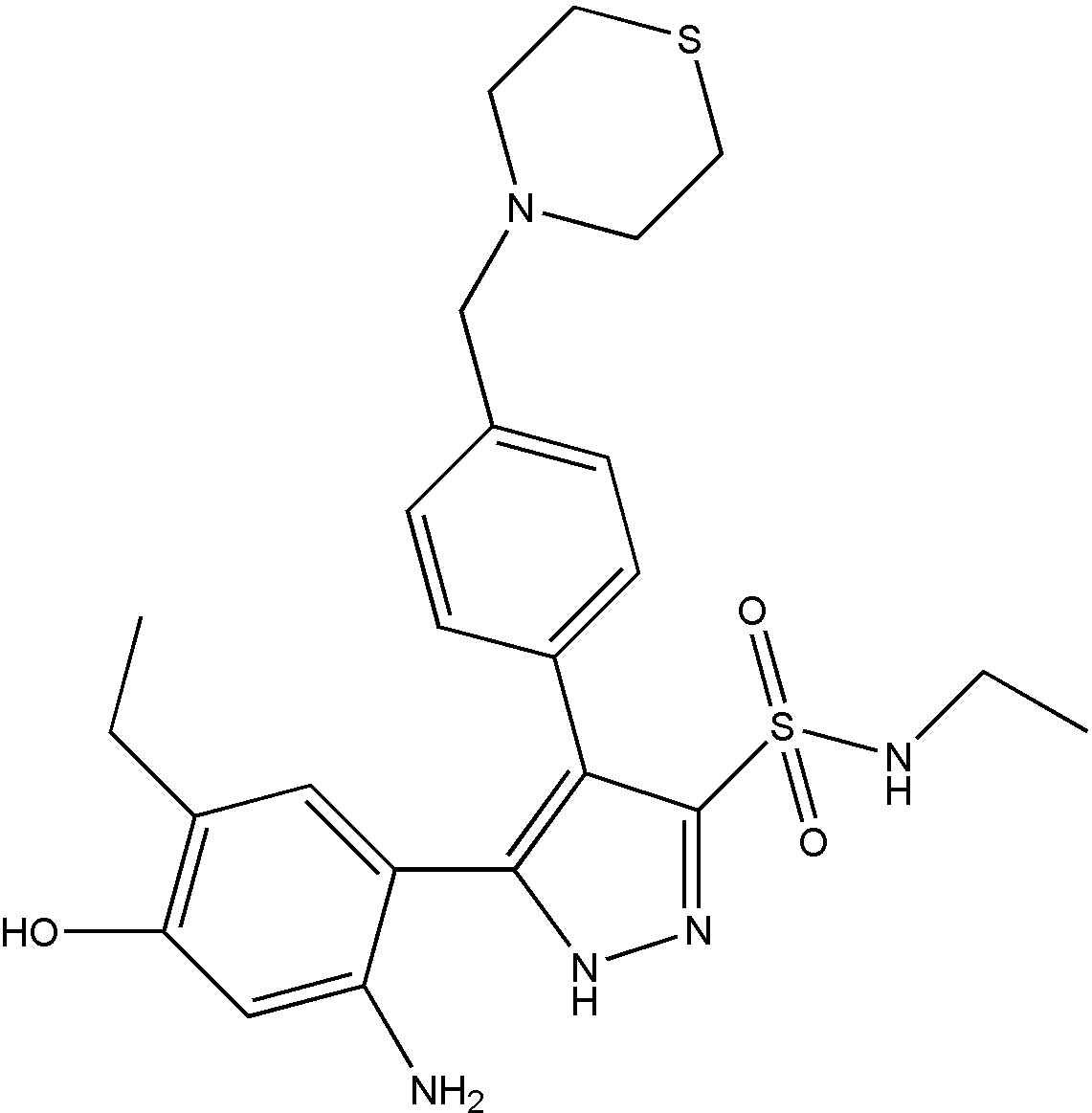 | 9.4 | ↑1.4 |
| A19 | 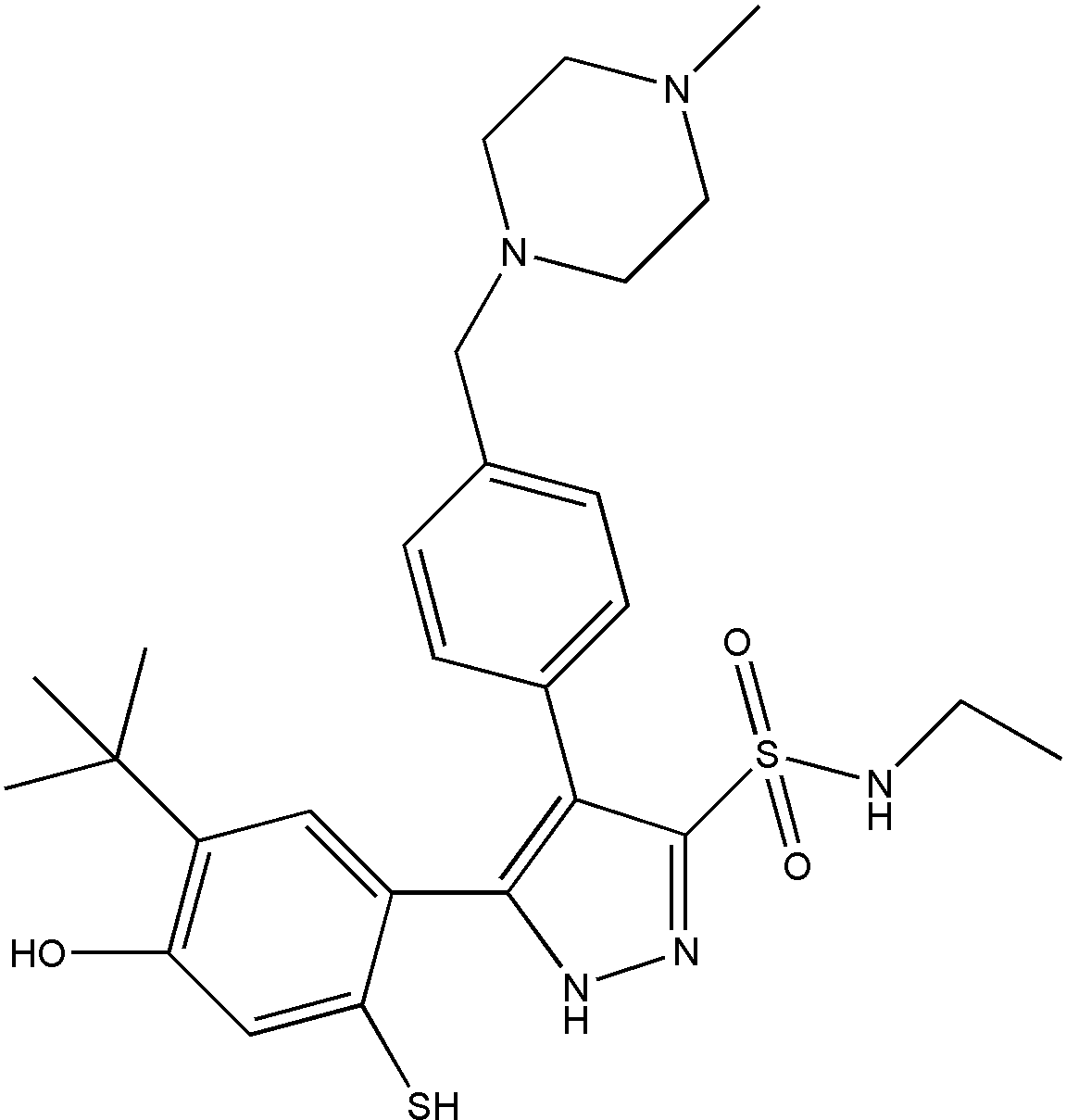 | 9.2 | ↑1.2 |
| A23 | 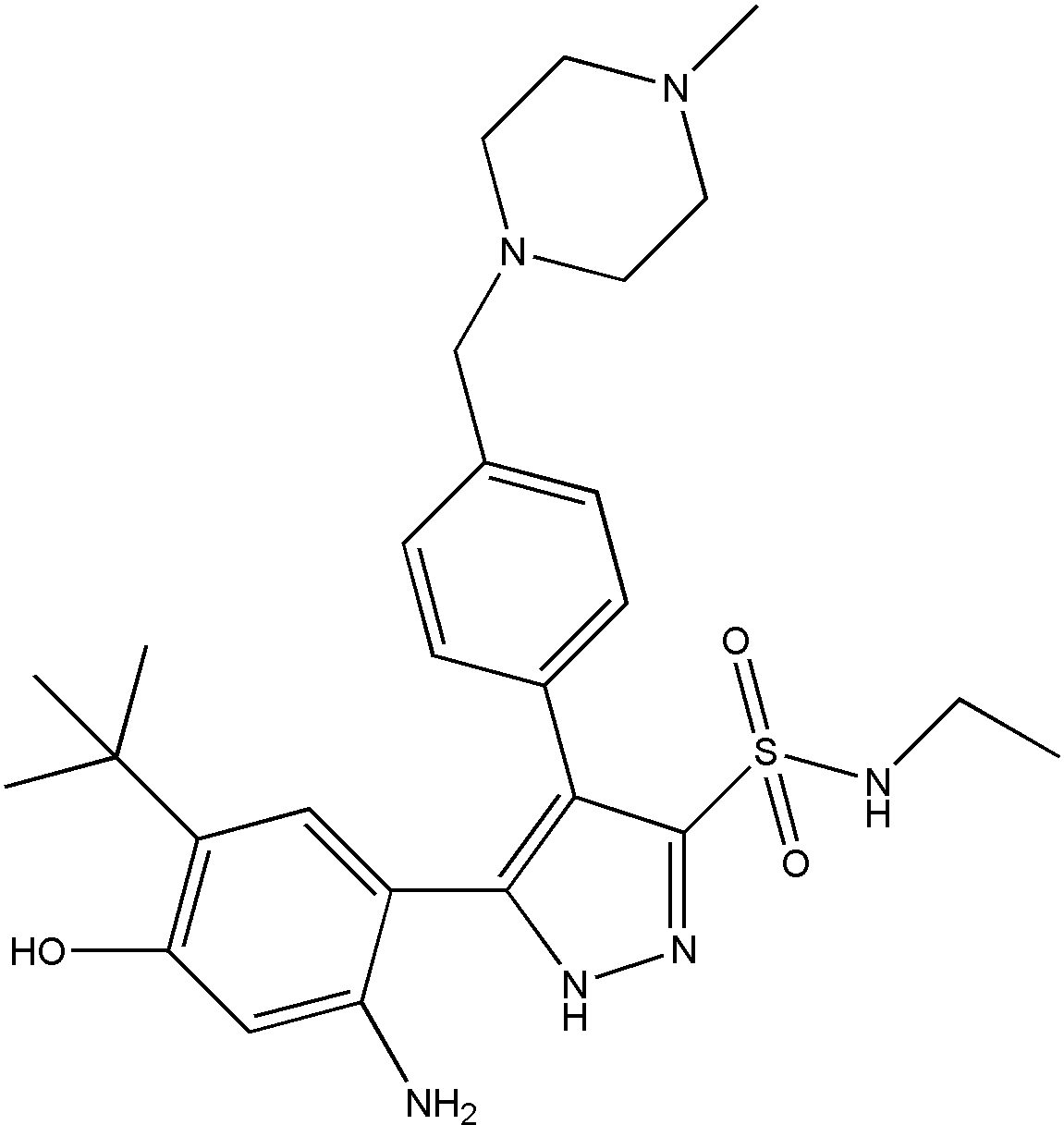 | 9.1 | ↑1.1 |
| A32 | 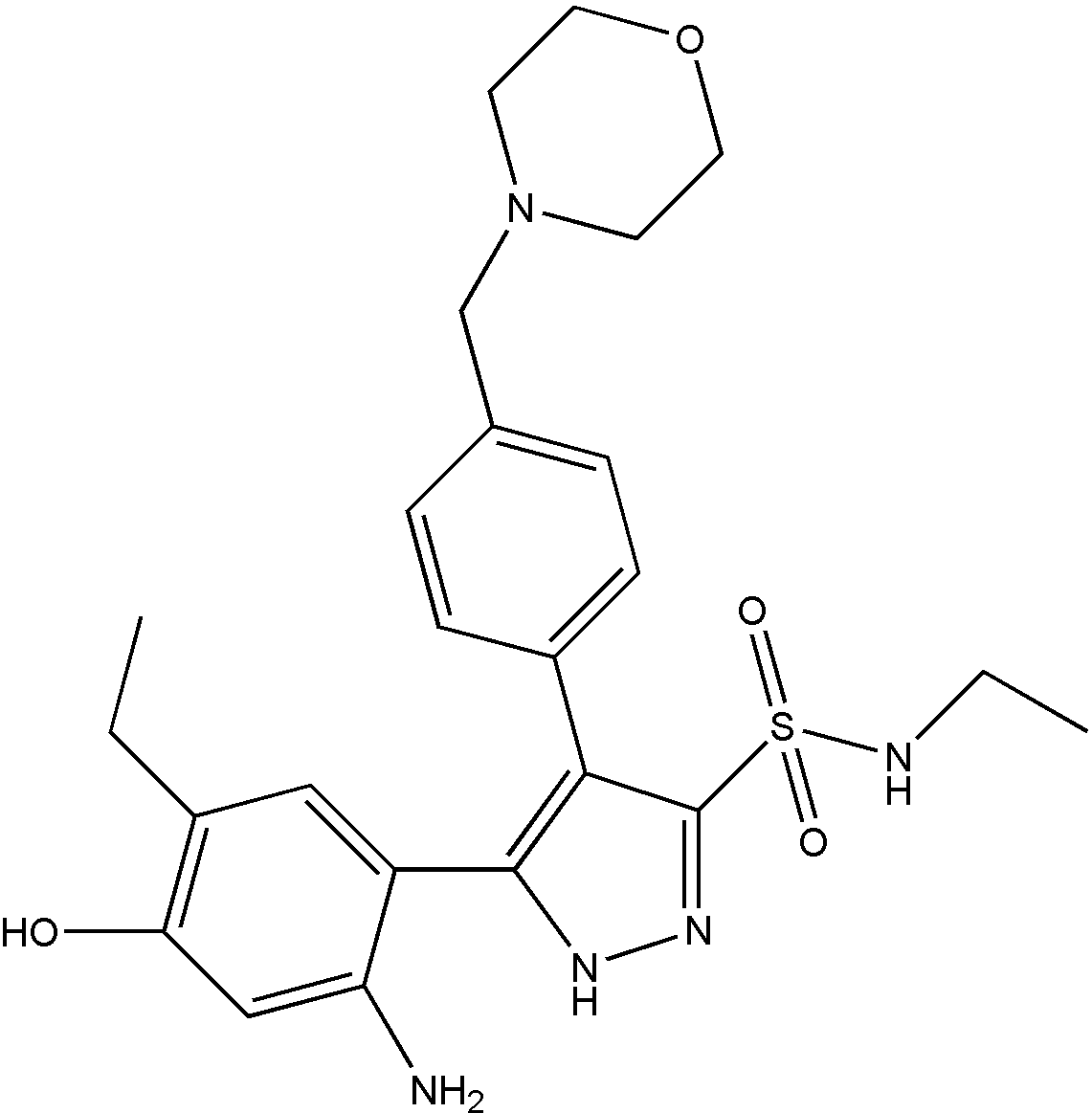 | 9.0 | ↑1.0 |
| A1 | 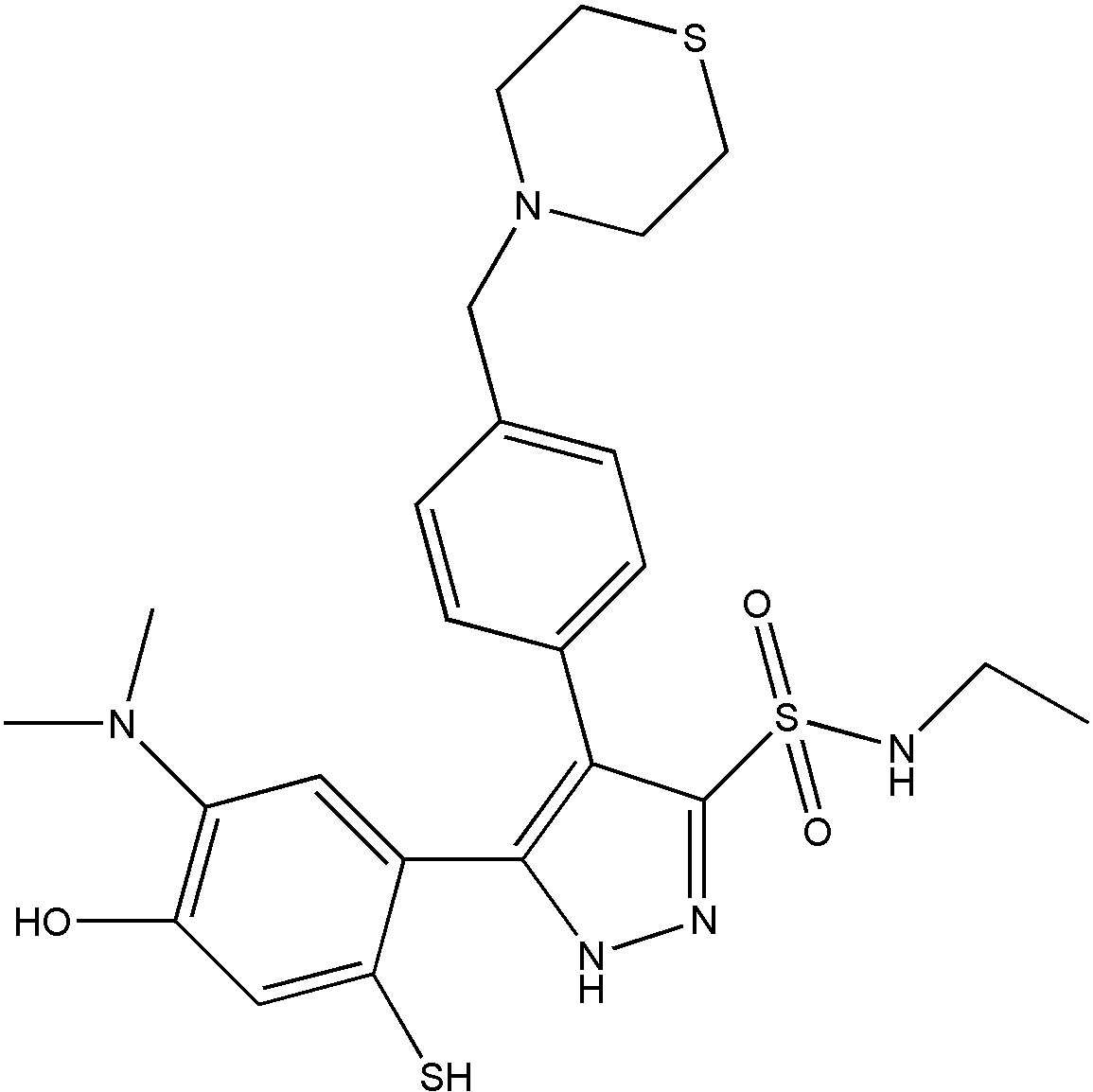 | 9.0 | ↑1.0 |
| A30 | 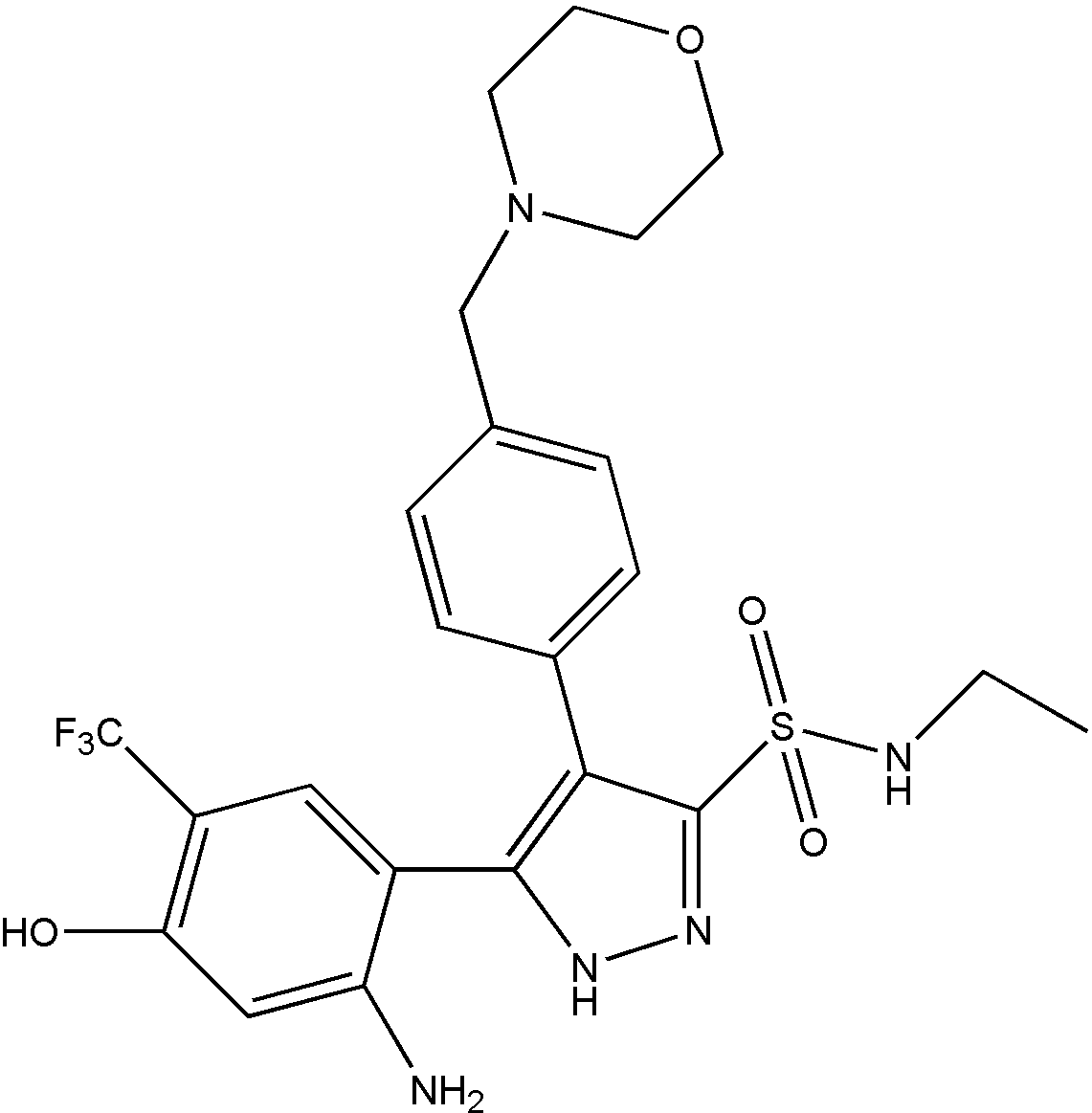 | 8.9 | ↑0.9 |
| A10 | 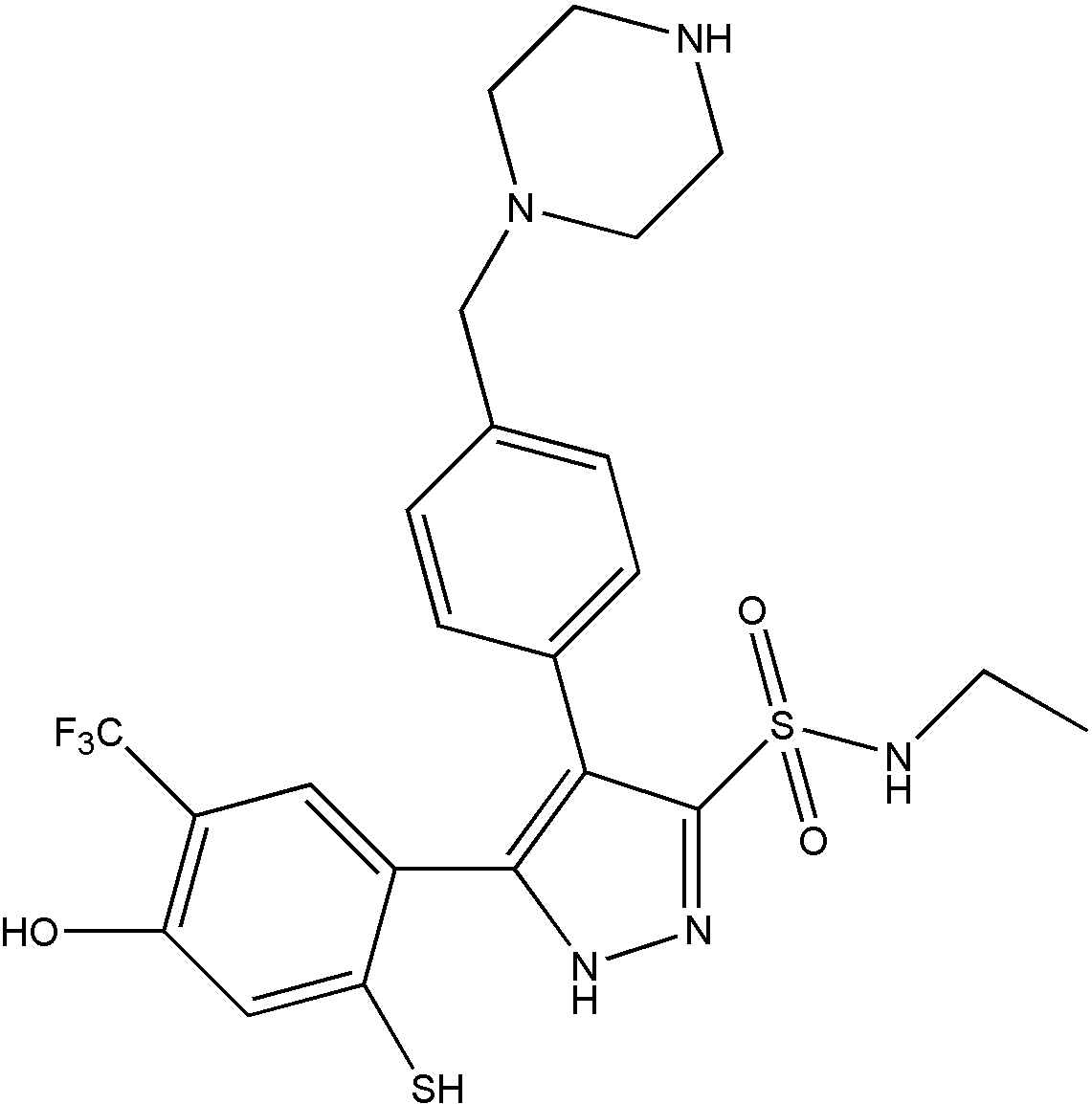 | 8.7 | ↑0.7 |
| A27 | 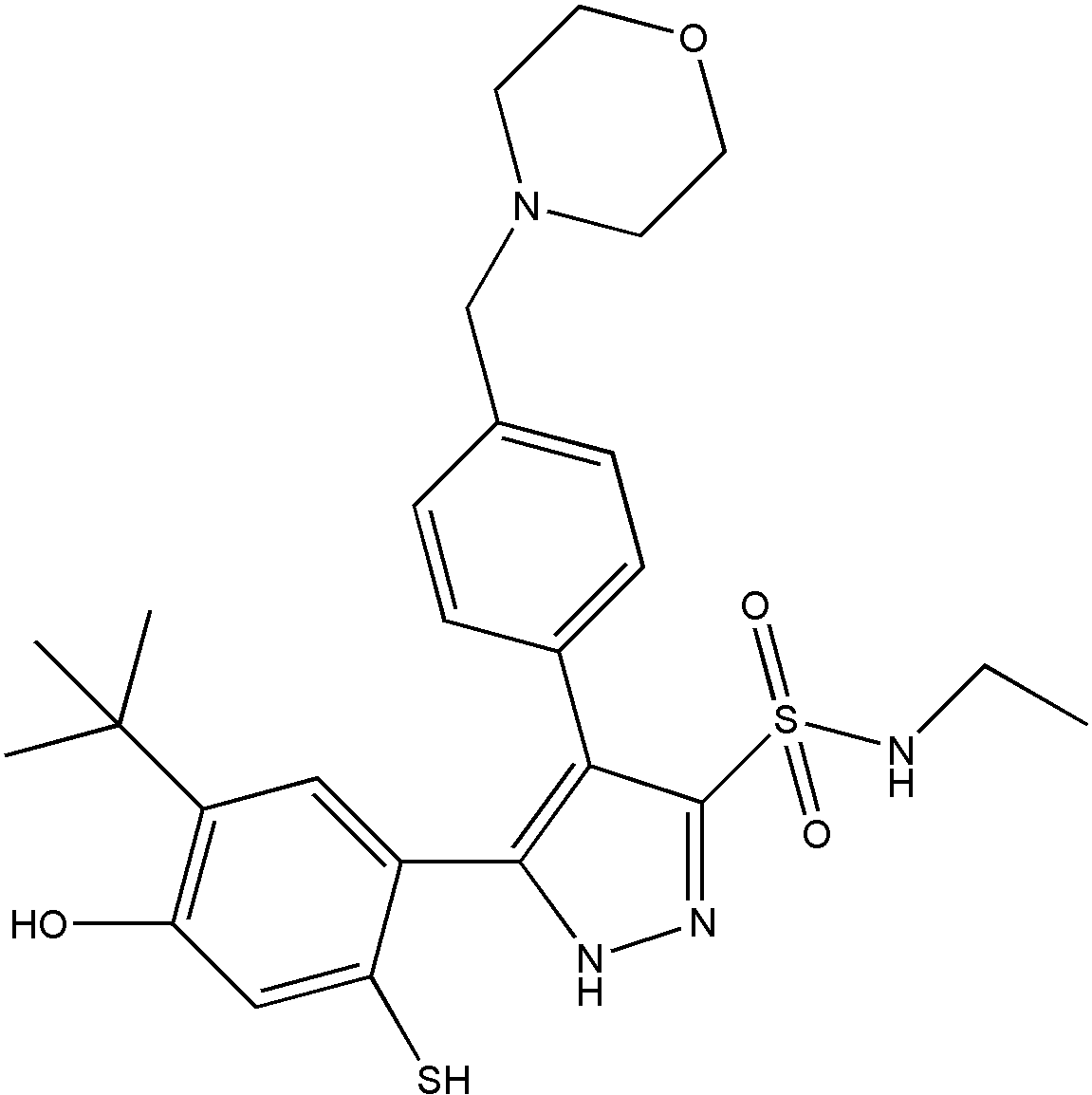 | 8.7 | ↑0.7 |
| A26 | 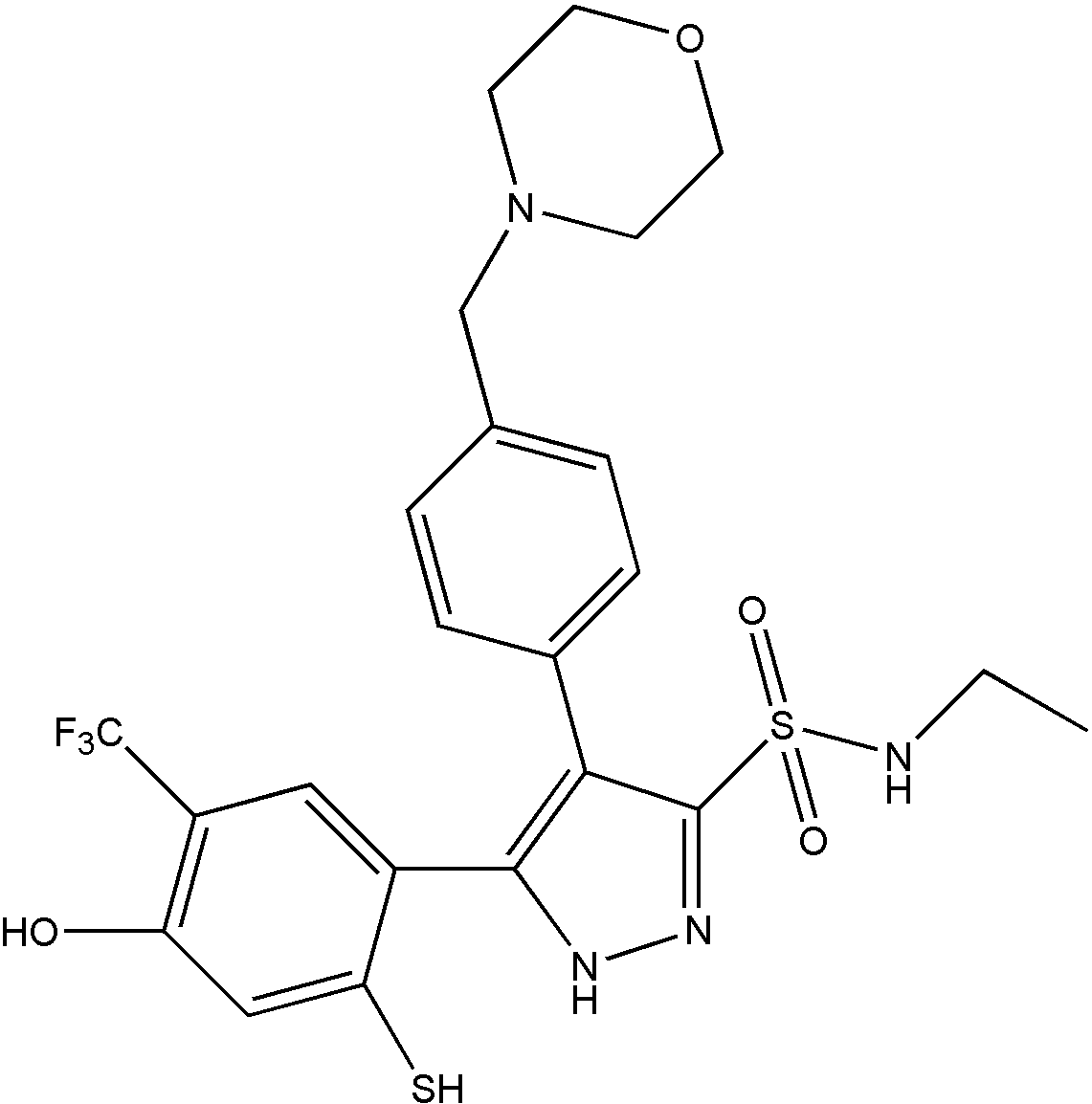 | 8.7 | ↑0.7 |
| A17 | 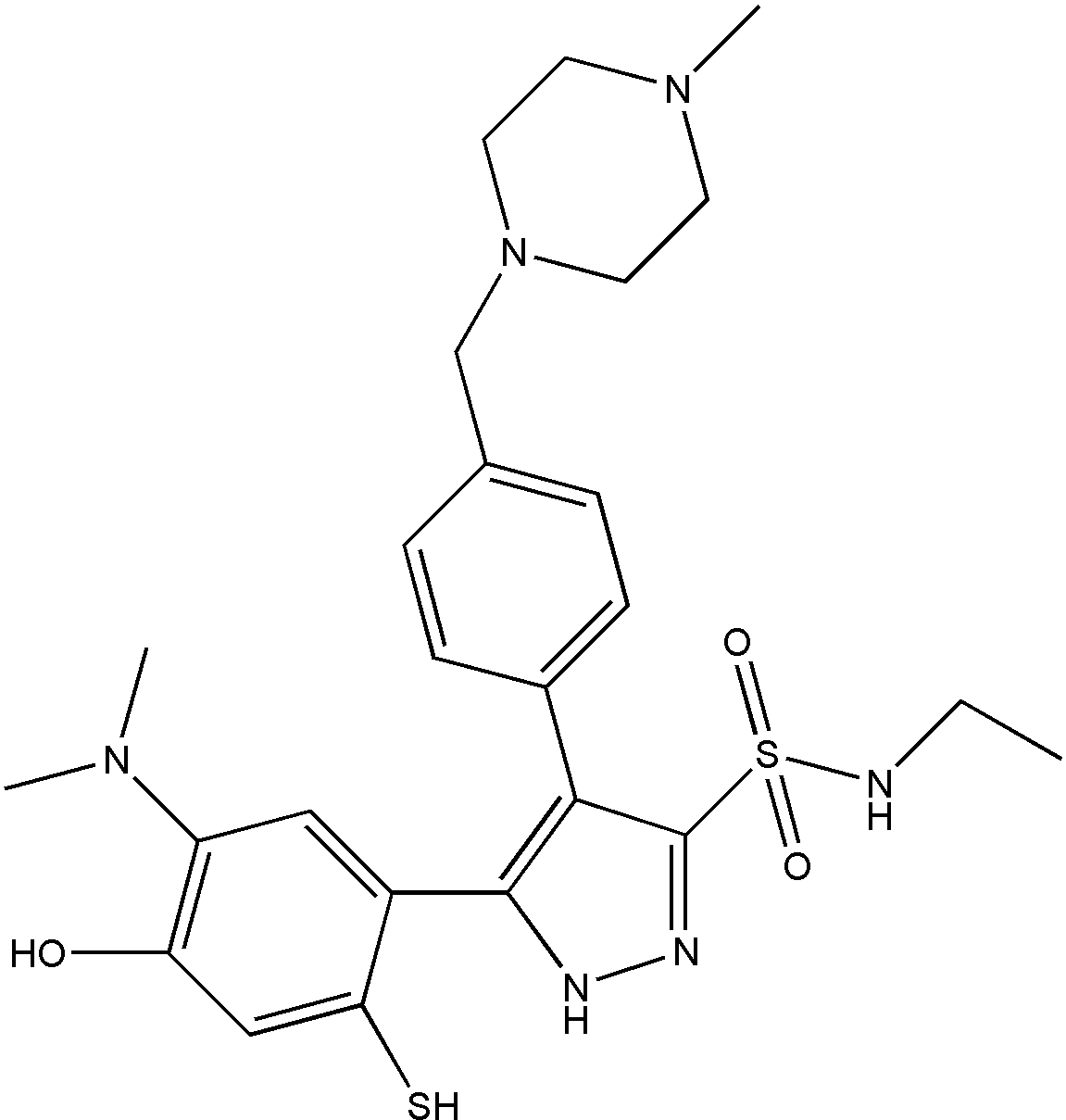 | 8.7 | ↑0.7 |
| A18 | 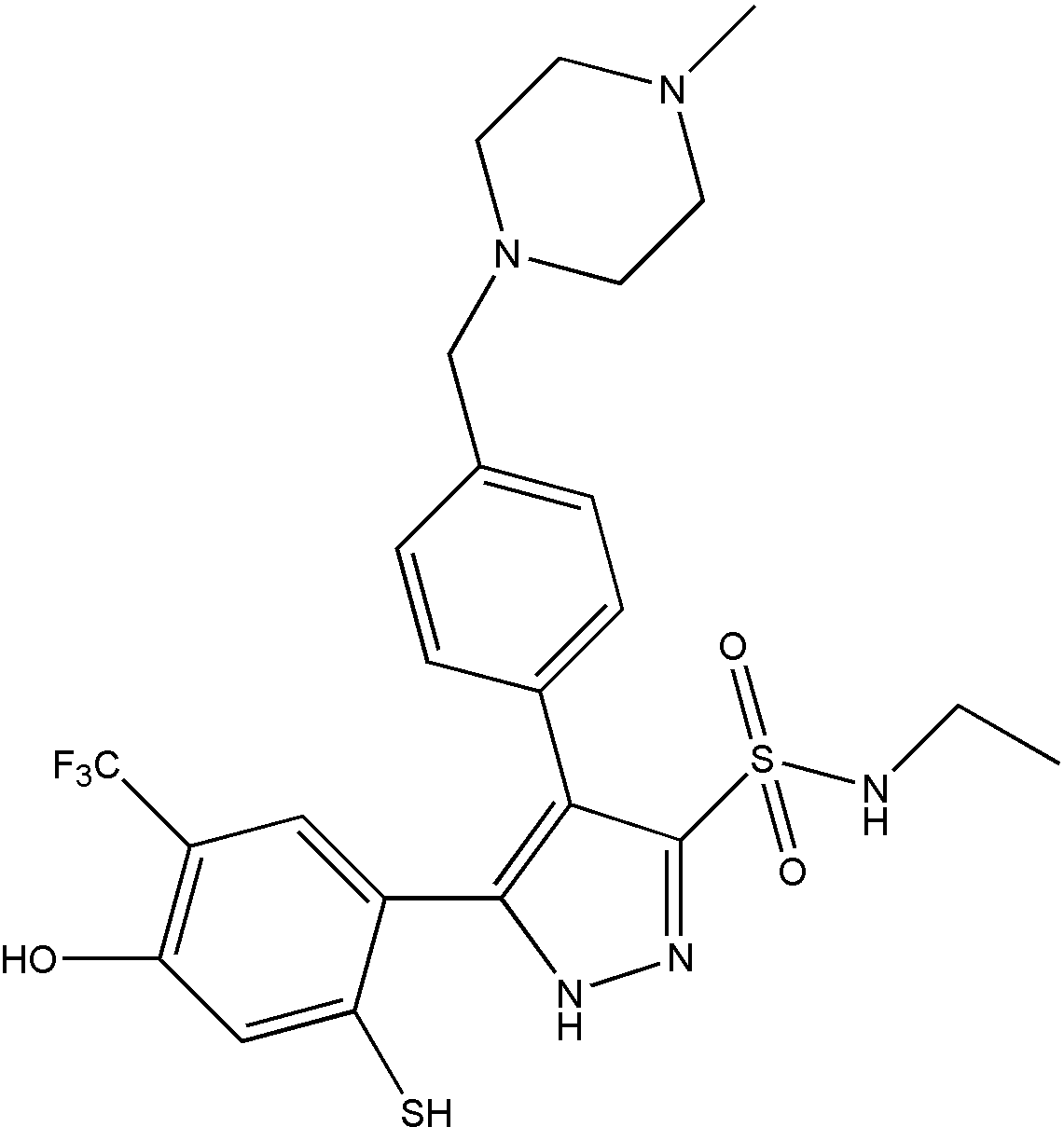 | 8.6 | ↑0.6 |
| A6 | 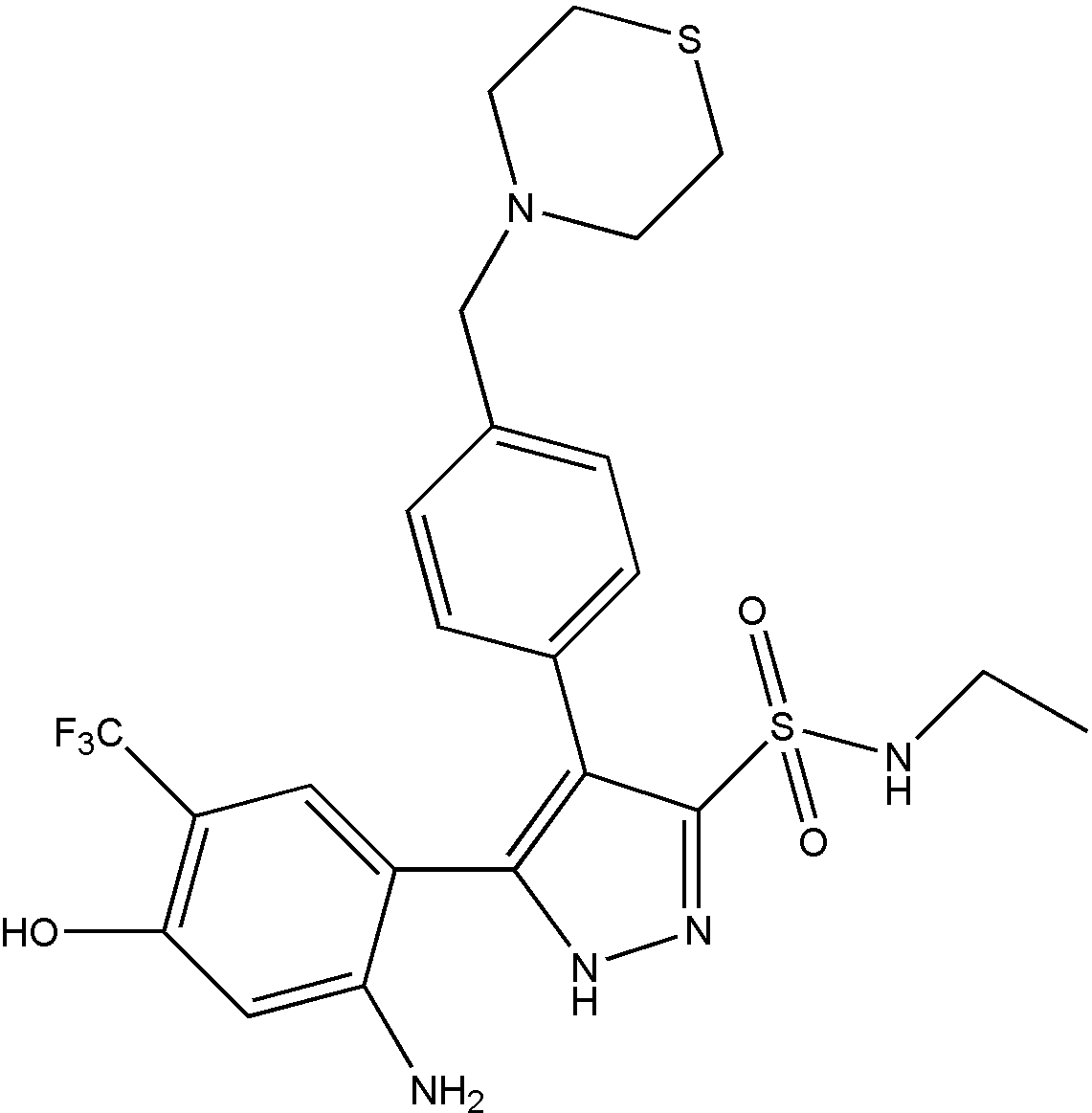 | 8.6 | ↑0.6 |
| A28 | 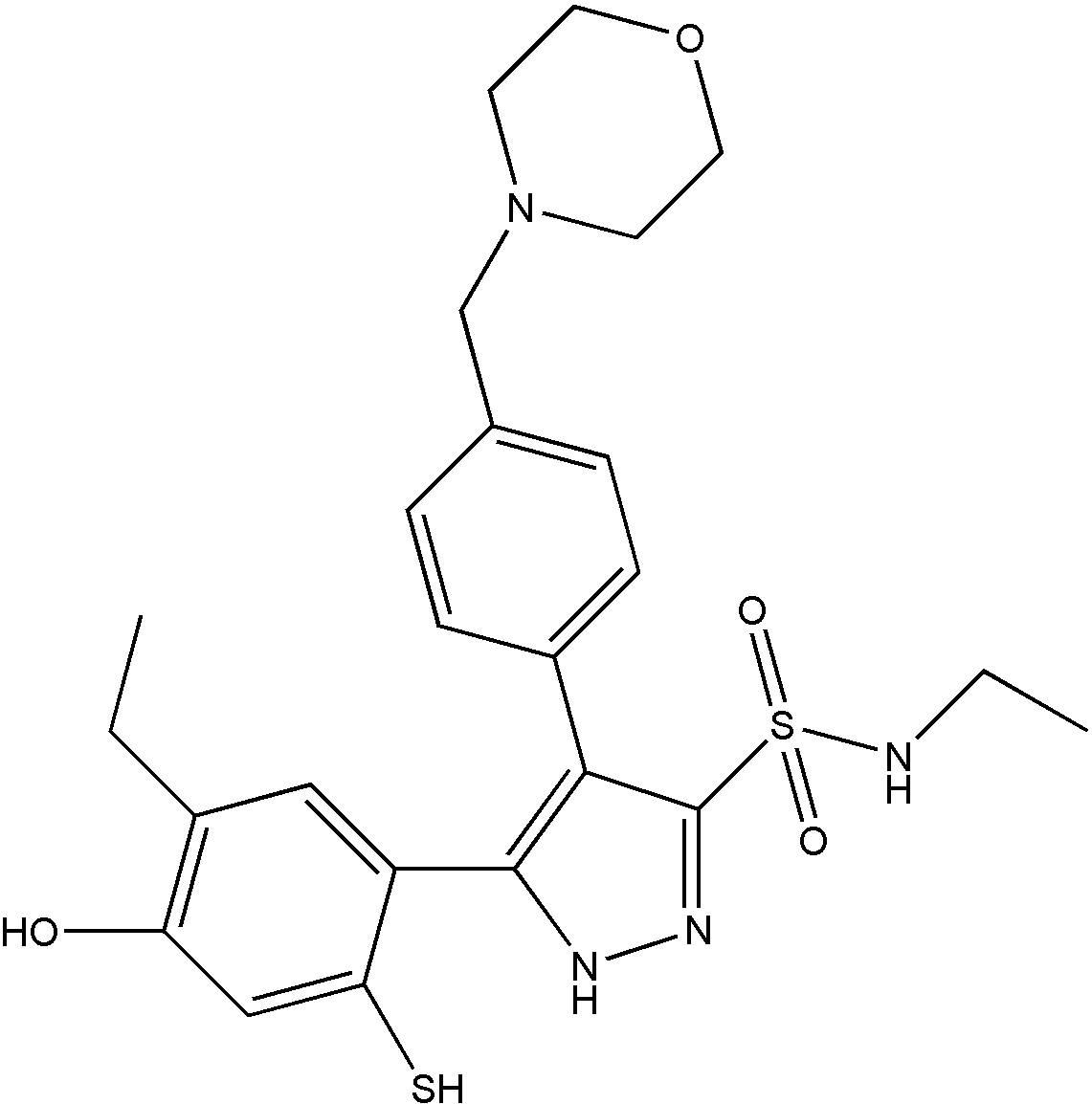 | 8.4 | ↑0.4 |
| A4 | 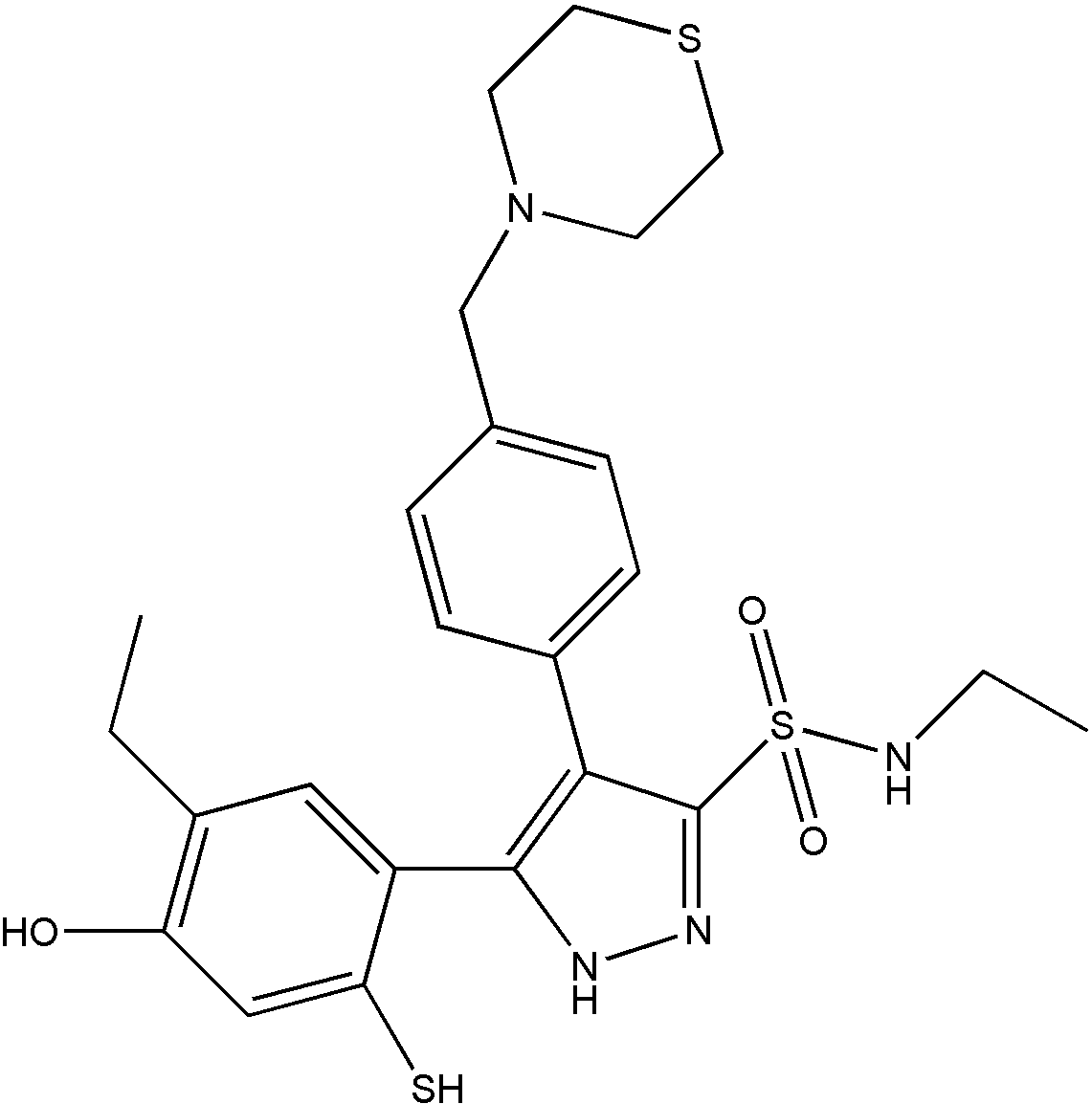 | 8.3 | ↑0.3 |
| A11 | 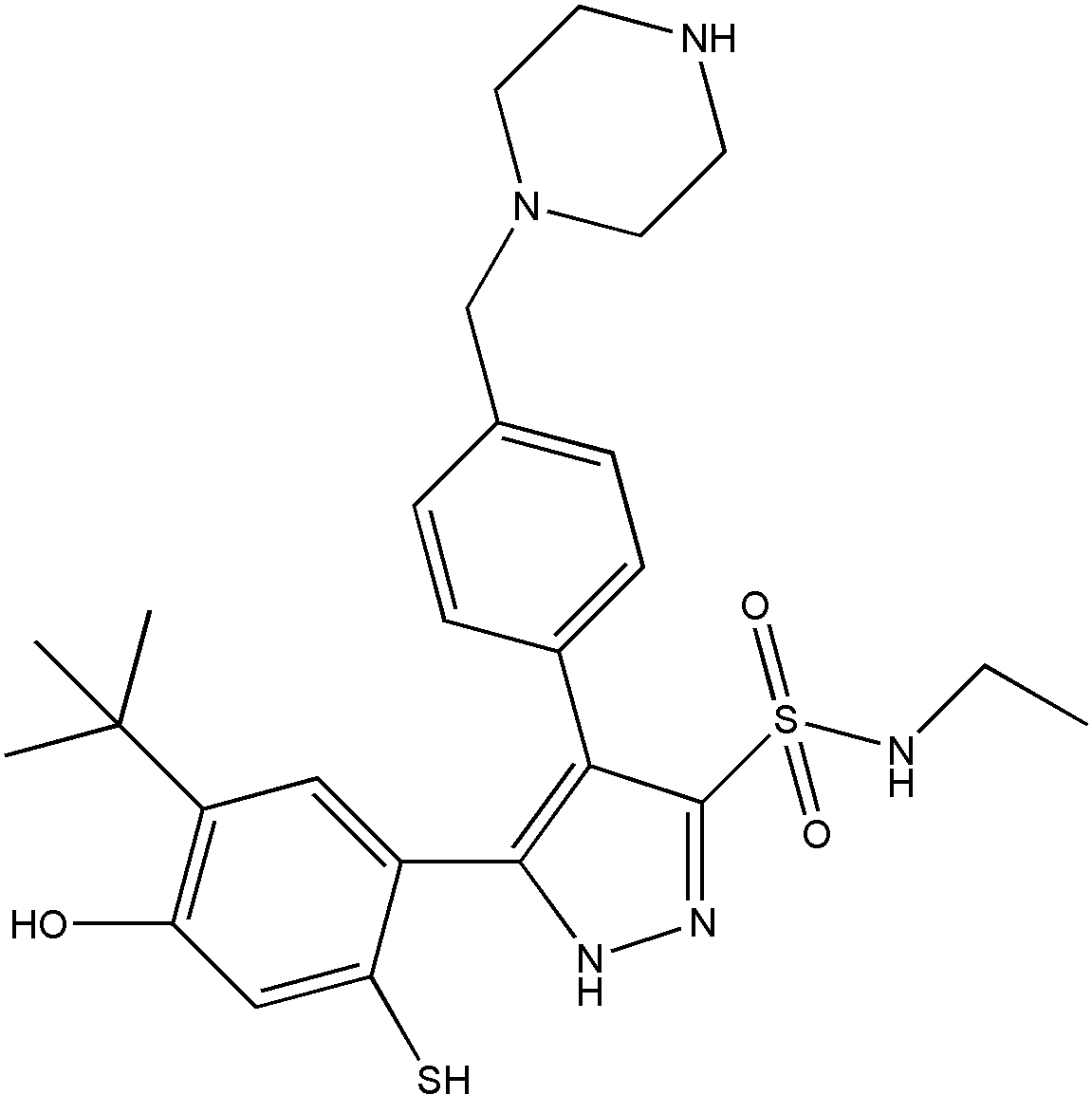 | 8.1 | ↑0.1 |
| A3 | 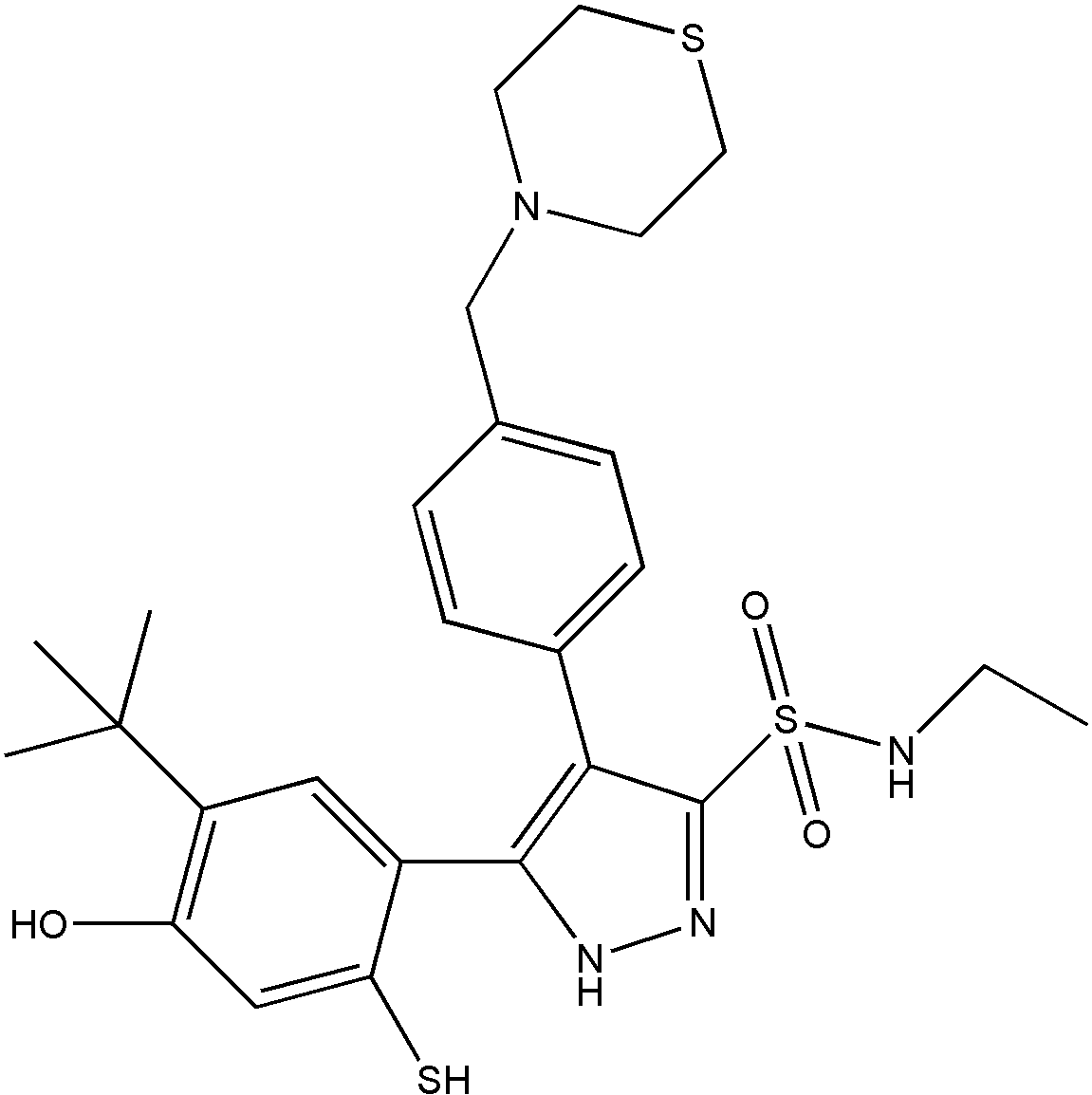 | 8.1 | ↑0.1 |
| A12 | 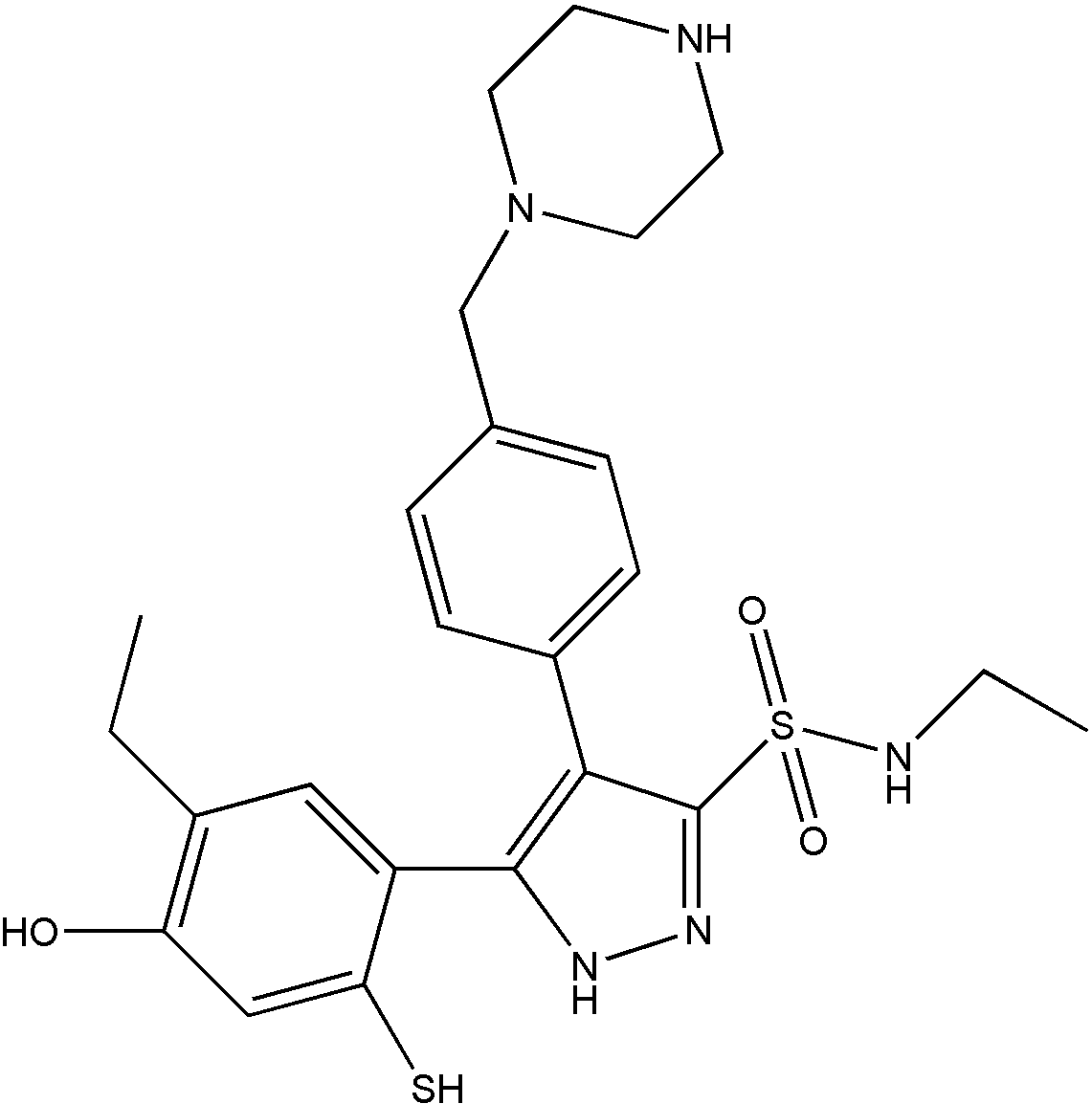 | 8.1 | ↑0.1 |
| A20 | 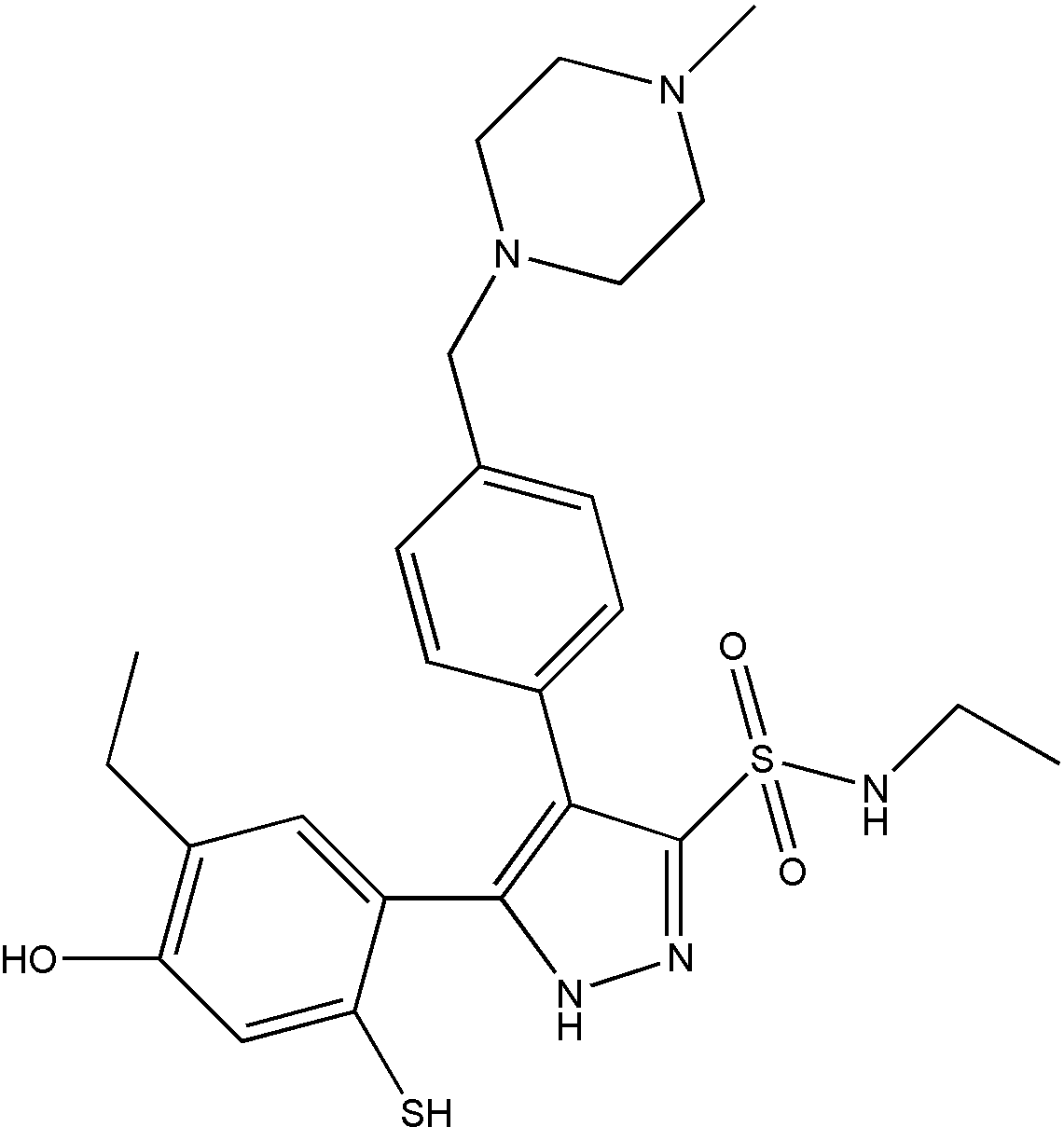 | 8.0 | 0.0 |
| A22 | 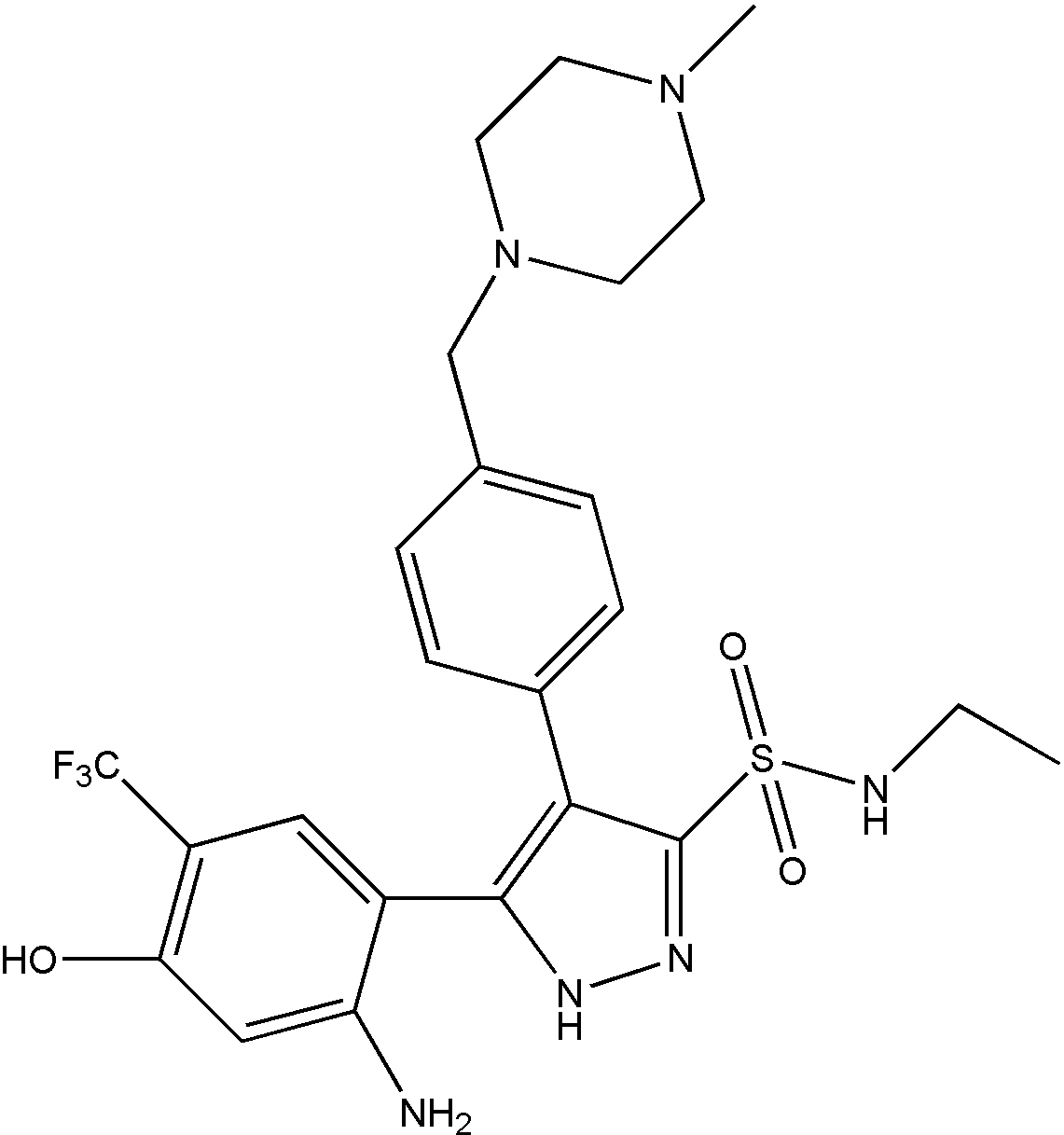 | 7.9 | ↓0.1 |
| A2 | 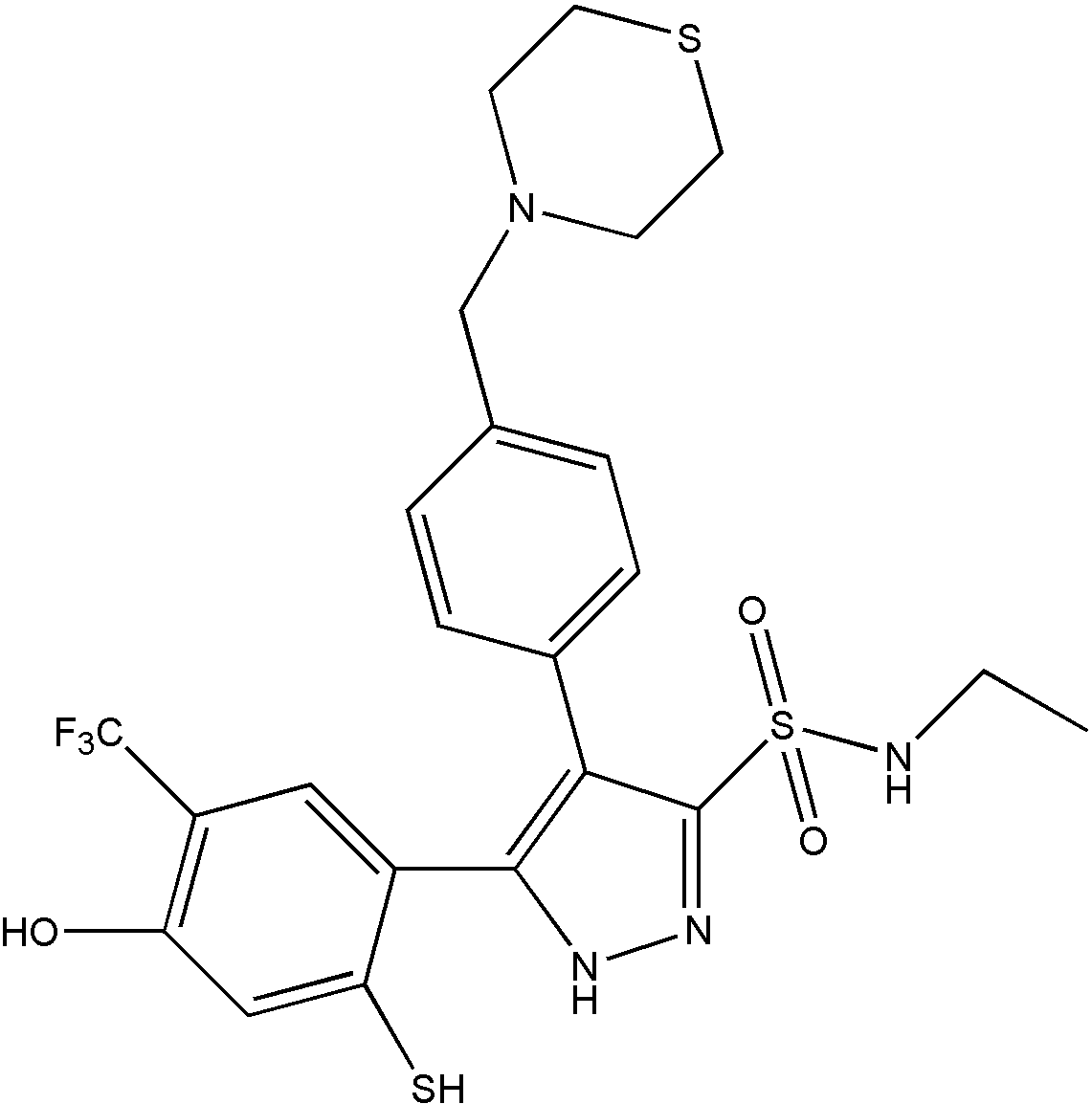 | 6.8 | ↓1.2 |
| A25 | 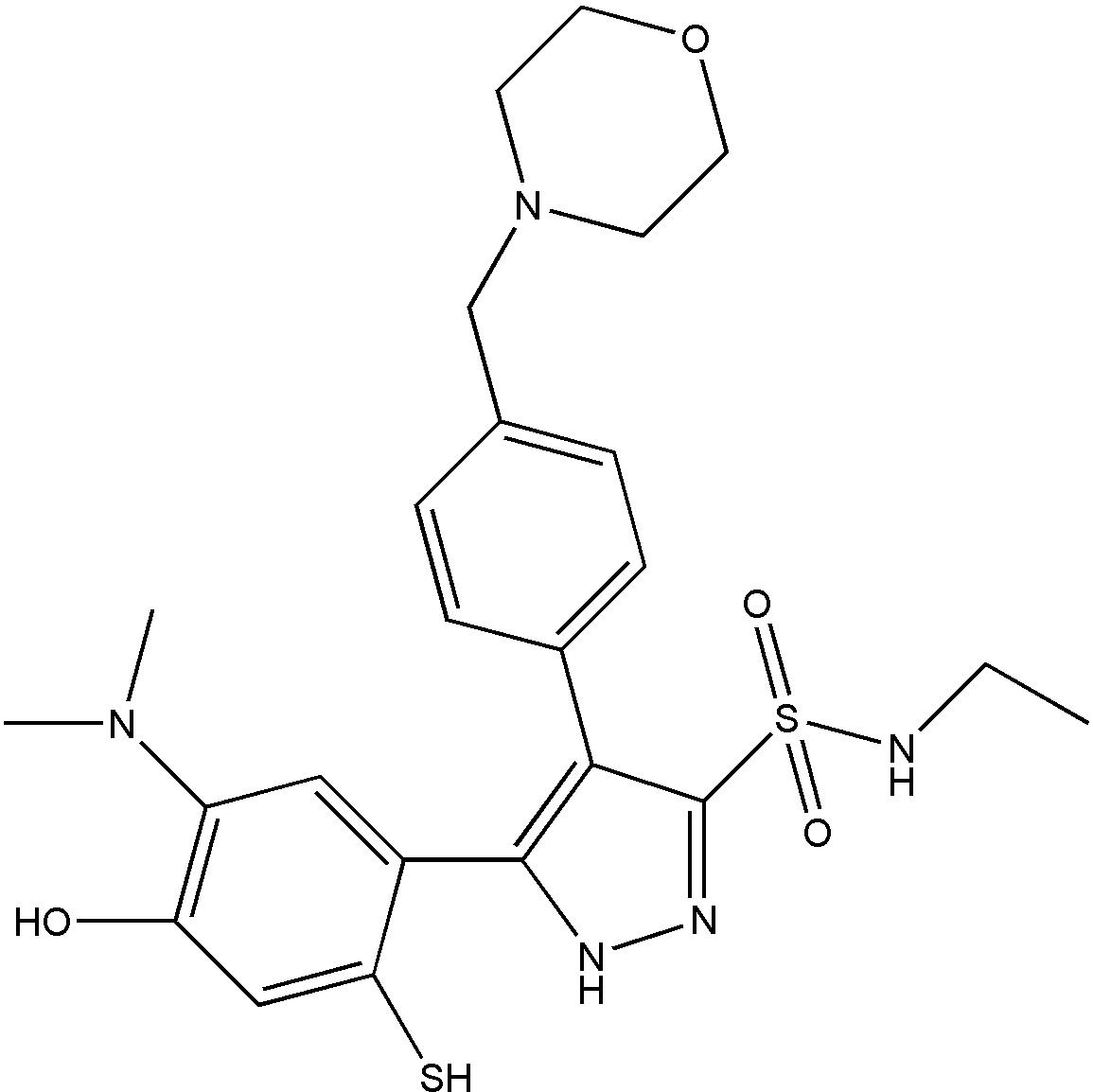 | 6.3 | ↓1.7 |

Annotation: The red means new derivatives with increased Total score values compared with NVP-AUY922 targeting to Hsp90^N^ determined by molecular docking with software SYBYL-X 2.0 and the blue means ones with decreased Total score values.
